# Genetic links between ovarian ageing, cancer risk and de novo mutation rates

**DOI:** 10.1038/s41586-024-07931-x

**Published:** 2024-09-11

**Authors:** Stasa Stankovic, Saleh Shekari, Qin Qin Huang, Eugene J. Gardner, Erna V. Ivarsdottir, Nick D. L. Owens, Nasim Mavaddat, Ajuna Azad, Gareth Hawkes, Katherine A. Kentistou, Robin N. Beaumont, Felix R. Day, Yajie Zhao, Hakon Jonsson, Thorunn Rafnar, Vinicius Tragante, Gardar Sveinbjornsson, Asmundur Oddsson, Unnur Styrkarsdottir, Julius Gudmundsson, Simon N. Stacey, Daniel F. Gudbjartsson, Breast Cancer Association Consortium, Kitale Kennedy, Andrew R. Wood, Michael N. Weedon, Ken K. Ong, Caroline F. Wright, Eva R. Hoffmann, Patrick Sulem, Matthew E. Hurles, Katherine S. Ruth, Hilary C. Martin, Kari Stefansson, John R. B. Perry, Anna Murray

**Affiliations:** 1 MRC Epidemiology Unit, Wellcome–MRC Institute of Metabolic Science, University of Cambridge, Cambridge, UK; 2 University of Exeter Medical School, University of Exeter, Exeter, UK; 3 School of Public Health, Faculty of Medicine, University of Queensland, Brisbane, Queensland Australia; 4 Wellcome Sanger Institute, Wellcome Genome Campus, Cambridge, UK; 5 deCODE Genetics/Amgen, Reykjavik, Iceland; 6 Centre for Cancer Genetic Epidemiology, Department of Public Health and Primary Care, University of Cambridge, Cambridge, UK; 7 DNRF Center for Chromosome Stability, Department of Cellular and Molecular Medicine, Faculty of Health and Medical Sciences, University of Copenhagen, Copenhagen, Denmark; 8 Department of Paediatrics, University of Cambridge, Cambridge, UK; 9 Metabolic Research Laboratory, Wellcome–MRC Institute of Metabolic Science, University of Cambridge, Cambridge, UK

**Keywords:** Genome-wide association studies, Quantitative trait loci, Endocrine reproductive disorders, Mutation, Rare variants

## Abstract

Human genetic studies of common variants have provided substantial insight into the biological mechanisms that govern ovarian ageing^1^. Here we report analyses of rare protein-coding variants in 106,973 women from the UK Biobank study, implicating genes with effects around five times larger than previously found for common variants (*ETAA1*, *ZNF518A*, *PNPLA8*, *PALB2* and *SAMHD1*). The *SAMHD1* association reinforces the link between ovarian ageing and cancer susceptibility^1^, with damaging germline variants being associated with extended reproductive lifespan and increased all-cause cancer risk in both men and women. Protein-truncating variants in *ZNF518A* are associated with shorter reproductive lifespan—that is, earlier age at menopause (by 5.61 years) and later age at menarche (by 0.56 years). Finally, using 8,089 sequenced trios from the 100,000 Genomes Project (100kGP), we observe that common genetic variants associated with earlier ovarian ageing associate with an increased rate of maternally derived de novo mutations. Although we were unable to replicate the finding in independent samples from the deCODE study, it is consistent with the expected role of DNA damage response genes in maintaining the genetic integrity of germ cells. This study provides evidence of genetic links between age of menopause and cancer risk.

## Abstract (web summary)

Analyses focusing on protein-truncating variants from 106,973 women from in the UK Biobank identify variants in genes that reinforce the link between reproductive lifespan in women and cancer risk in both sexes.

## Main

Reproductive longevity in women varies substantially in the general population and has profound effects on fertility and health outcomes in later life^1,2^. Women are born with a non-renewable ovarian reserve, which is established during fetal development. This reserve is continuously depleted throughout reproductive life, ultimately leading to menopause^3^. Variation in menopause timing is largely dependent on the differences in the size of the initial oocyte pool and the rate of follicle loss. Natural fertility is believed to be closely associated with menopause timing, and it declines on average ten years before the onset of menopause^4^. The effect of early menopause on infertility is becoming increasingly relevant owing to the secular trend of delaying parenthood to later maternal age at childbirth, especially in Western countries. In addition, normal variation in reproductive lifespan is causally associated with the risk of a wide range of disease outcomes, such as type 2 diabetes mellitus, cancer and impaired bone health, further highlighting the need for better understanding of the regulators and physiological mechanisms involved in reproductive ageing^1^.

The variation in timing of menopause reflects a complex mix of genetic and environmental factors that population-based studies have begun to unravel. Previous genome-wide association studies (GWAS) have successfully identified around 300 distinct common genomic loci associated with the timing of menopause^1^. These reported variants cumulatively explain 10–12% of the variance in age at natural menopause (ANM) and 31–38% of the overall estimated single nucleotide polymorphism (SNP) heritability^1,5,6^. Two-thirds of the GWAS signals implicate genes that regulate DNA damage response (DDR), highlighting the particular sensitivity of oocytes to DNA damage due to the prolonged state of cell cycle arrest across the lifetime^1,7–13^. Genetic studies for ANM to date have focussed largely on assessing common genetic variation, with little insight into the role of rarer, protein-coding variants. Initial whole-exome sequencing (WES) analyses in the UK Biobank identified gene-based associations with ANM for *CHEK2*, *DCLRE1A*, *HELB*, *TOP3A*, *BRCA2* and *CLPB*^1,5^. Here we aimed to explore the role of rare damaging variants in ovarian ageing in greater detail through a combination of enhanced phenotype curation, better-powered statistical tests and assessment of different types of variant class at lower allele frequency thresholds (Supplementary Information). Using these approaches, we identify five genes harbouring variants with large effects, highlighting *ZNF518A* as a major transcriptional regulator of ovarian ageing. Furthermore, we extend these observations to provide initial evidence that women at increased genetic risk of earlier menopause have increased rates of de novo mutations in their offspring.

## Exome-wide gene burden associations

**Fig. 1:**
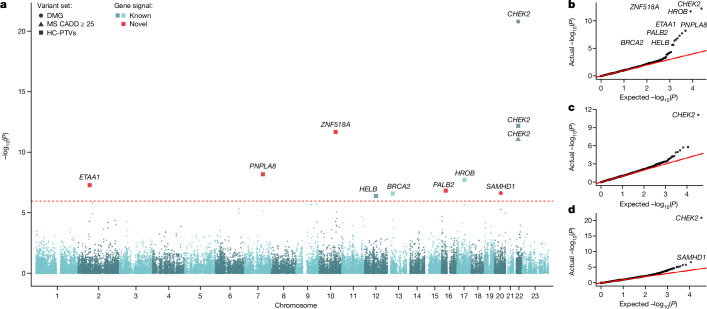
Exome-wide associations with ANM. **a**, Manhattan plot showing gene burden test results for ANM from BOLT-LMM in 106,973 female participants. Genes passing exome-wide significance (*P* < 1.08 × 10^−6^) are indicated, with the shape signifying the variant class tested and colour indicating the novelty. MS, missense. **b**–**d**, QQ plots of *P* values from BOLT-LMM against expected *P* values for high-confidence PTVs: *λ* = 1.047 (**b**), CADD ≥ 25 missense variants: *λ* = 1.050 (**c**) and damaging variants: *λ* = 1.050 (**d**).

**Extended Data Fig. 1:**
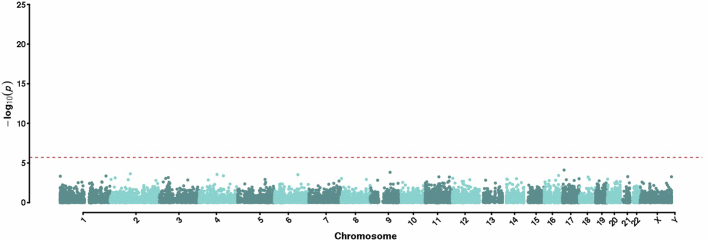
Exome-wide association results for synonymous variants. Plotted are per-gene burden results for synonymous variants. The red line indicates the exome-wide significant P value after Bonferroni correction of 1.08*10^−6^.

Previous studies have focused largely on assessing the role of common genetic variation on ovarian ageing. We sought to better understand the role of rare coding variation in ovarian ageing using WES data available in 106,973 post-menopausal female UK Biobank participants of European genetic ancestry^14^. We conducted individual gene burden association tests by collapsing genetic variants according to their predicted functional categories. We defined three categories of rare exome variants with minor allele frequency (MAF) < 0.1%: high-confidence protein-truncating variants (HC-PTVs), missense variants with combined annotation-dependent depletion (CADD) score ≥ 25, and ‘damaging’ variants (DMG, defined as combination of HC-PTVs and missense variants with CADD ≥ 25). We analysed 17,475 protein-coding genes with a minimum of 10 rare allele carriers in at least one of the masks tested. The primary burden association analysis was conducted using BOLT-LMM^15^ (Fig. 1 and Supplementary Table 1). The low exome-wide inflation scores (Fig. 1b–d) and the absence of significant association with synonymous variant burden for any gene indicate that our statistical tests are well calibrated (Extended Data Fig. 1).

**Fig. 2:**
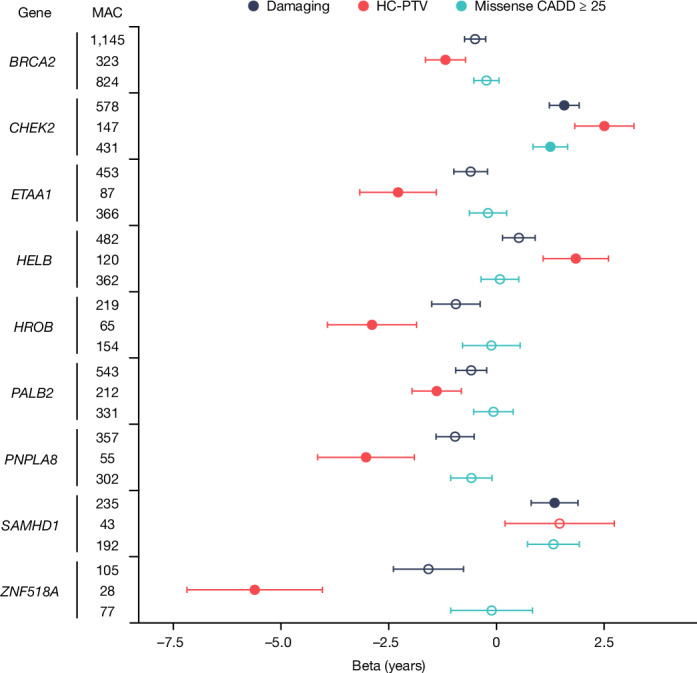
Forest plot for gene burden associations with ANM. Exome-wide significant (*P* < 1.08 × 10^−6^) genes (filled circles) are displayed; an unfilled circle indicates a nonsignificant association. Points and error bars indicate beta and 95% CI, respectively, for the indicated variant category. Beta values, CIs, minor allele counts (MACs) and *P* values are derived from BOLT-LMM (values are given in Supplementary Table 2). *n* = 115,051 individuals with ANM are included in the analysis.

**Extended Data Fig. 2:**
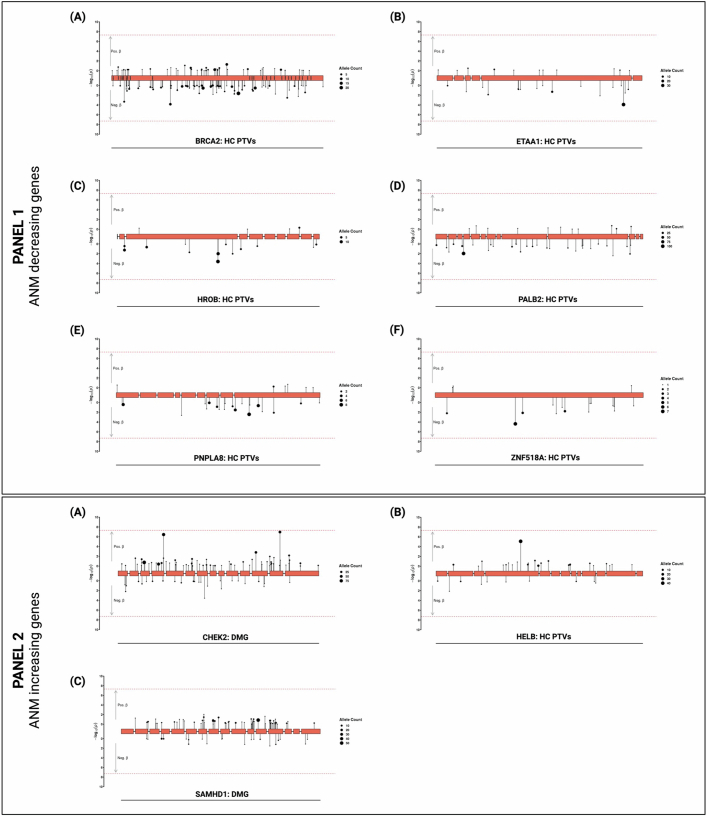
Lollipop plots show the variants, clustered for the best performing functional mask in a gene, which went into the gene burden test for ANM using BOLT-LMM. The arrows pointing upwards represent the variants positively associated with ANM, while the ones pointing downwards show the negatively associated variants. The size of the point indicates the allele count in carriers. **Panel 1: Variant level associations for ANM decreasing WES genes. (A)** *BRCA2* HC PTV mask (genomic size = 84,761 bp, coding sequence = 10,254 bp); **(B)** *ETAA1*, HC PTV mask (genomic size = 14,757 bp, coding sequence = 2,778 bp); **(C)** *HROB*, HC PTV mask (genomic size = 20,547 bp, coding sequence = 1,938 bp); **(D)** *PALB2*, HC PTV mask (genomic size = 38,146 bp, coding sequence = 3,558 bp); **(E)** *PNPLA8*, HC PTV mask (genomic size = 55,719 bp, coding sequence = 2,346 bp); and **(F)** *ZNF518A*, HC PTV mask (genomic size = 33,463 bp, coding sequence = 4,449 bp). **Panel 2: Variant level associations for ANM increasing WES genes. (A)** *CHEK2*, damaging mask (genomic size = 54,078 bp, coding sequence = 1,629 bp); **(B)** *HELB*, HC PTV mask (genomic size = 35,707 bp, coding sequence = 3,261 bp); and **(C)** *SAMHD1*, damaging mask (genomic size = 61,480 bp, coding sequence = 1,878 bp).

We identified rare variation in nine genes associated with ANM at exome-wide significance (*P* < 1.08 × 10^−6^; Figs. 1 and 2, Extended Data Fig. 2 and Supplementary Tables 1 and 2). These were confirmed by an independent group of analysts using different quality control and analysis pipelines (Supplementary Tables 1 and 2). Three of these genes have been previously reported in UK Biobank WES analyses^5^, and we confirm the associations of *CHEK2* (beta = 1.57 years (95% confidence interval (CI): 1.23–1.92), *P* = 1.6 × 10^−21^, *n* = 578 damaging allele carriers) and *HELB* (beta = 1.84 years (95% CI: 1.08–2.60), *P* = 4.2 × 10^−7^, *n* = 120 HC-PTV carriers) with later ANM and a previously borderline association of *HROB* with earlier ANM (beta = −2.89 years (95% CI: 1.86–3.92), *P* = 1.9 × 10^−8^, *n* = 65 HC-PTV carriers). In addition, our previous ANM GWAS analyses^1^ identified an individual low-frequency PTV variant in *BRCA2*, which we now extend to demonstrate that, in aggregate, *BRCA2* HC-PTV carriers exhibit 1.18 years earlier ANM (beta = −1.18 years (95% CI: 0.72–1.65), *P* = 2.6 × 10^−7^, *n* = 323). Rare variants in the remaining five genes (*ETAA1*, *ZNF518A*, *PNPLA8*, *PALB2* and *SAMHD1*) have not previously been implicated in ovarian ageing. Effect sizes of these associations range from 5.61 years earlier ANM for HC-PTV carriers in *ZNF518A* (95% CI: 4.04–7.18, *P* = 2.1 × 10^−12^, *n* = 28), to 1.35 years later ANM for women carrying damaging alleles in *SAMHD1* (95% CI: 0.81–1.89, *P* = 2.4 × 10^−7^, *n* = 235). This contrasts with a maximum effect size of 1.06 years (median 0.12 years) for common variants (MAF > 1%) identified by previous ANM GWAS^1^.

We next attempted to replicate these findings using two independent datasets. First, from the Icelandic deCODE study^16,17^. Despite the substantially smaller sample size (*n* = 27,678 women with ANM), rarity of the alleles we were testing, and minor differences in allele frequency and variant classification, we observed consistent effect estimates for all nine genes that we identified (Supplementary Table 3). This included nominally significant associations at *BRCA2*, *CHEK2*, *ETAA1*, *HROB*, *HELB*, *SAMHD1* and *ZNF518A*. Second, we used data in up to 26,258 women with ANM from the BRIDGES study^18^. As this study used a targeted sequencing approach of suspected breast cancer genes, it was informative for only *BRCA2*, *PALB2* and *CHEK2*. Despite the small sample size, for each of these genes we found effect estimates consistent with our discovery analyses, which were maintained when adjusting for cancer status and within women not diagnosed with breast cancer (Supplementary Table 4). Notably, we replicated the novel association with *PALB2*, where the 78 women carrying PTVs experienced menopause 1.78 years earlier on average (*P* = 4.6 × 10^−4^). Differences in allele frequency cut-offs had minimal effect on variants included in burden tests, because we only tested predicted deleterious variants and these were mostly rare (less than 0.1%).

We next sought to understand why previous analyses of UK Biobank WES data missed the associations that we report here, and conversely why we did not identify associations with other previously reported genes. Of the seven genes identified by Ward et al.^5^, three were also identified by our study (*CHEK2*, *HELB* and *HROB*), three were recovered when we increased our burden test MAF threshold from 0.1% to 1% (*DCLRE1A*, *RAD54L* and *TOP3A*), and an additional gene fell just below our *P* value threshold when considering variants with <1% MAF (*CLPB*; *P* = 1.2 × 10^−^^5^). By contrast, our identification of novel associations that were not reported by Ward et al. (*BRCA2*, *ETAA1*, *PALB2*, *PNPLA8*, *SAMHD1* and *ZNF518A*) is probably explained by differences in phenotype preparation, sample size, variant annotation and the statistical model used (see Supplementary Information and Supplementary Table 5).

## Overlap with common variant associations

To explore the overlap between common and rare variant association signals for ANM, we integrated our exome-wide results with data generated from the largest reported common variant GWAS of ANM^1^.

Five of our nine identified WES genes (*CHEK2*, *BRCA2*, *ETAA1*, *HELB* and *ZNF518A*) mapped within 500 kb of a common GWAS signal (Supplementary Table 6). Notably, we previously reported a common, predicted benign, missense variant (rs35777125-G439R, MAF = 11%) in *ETAA1* associated with 0.26 years earlier ANM^1^. By contrast, our WES analysis showed that carriers of rare HC-PTVs in *ETAA1* show a nearly 10-fold earlier ANM (beta = −2.28 years (95% CI: 1.39–3.17), *P* = 5.30 × 10^−8^, *n* = 87). Furthermore, three independent non-coding common GWAS signals around 150 kb apart (MAF: 2.8–47.5%, beta: −0.28 to 0.28 years per minor allele) were reported proximal to *ZNF518A*, whereas gene burden testing finds that rare HC-PTV carriers show nearly 20-fold earlier ANM than common variant carriers (beta = −5.61 years (95% CI: 4.04–7.18), *P* = 2.10 × 10^−12^, *n* = 28). *ZNF518A* is a poorly characterized C2H2 zinc-finger transcription factor, which has been shown to associate with PRC2 and G9A–GLP repressive complexes along with its paralogue *ZNF518B*, suggesting a potential role in transcriptional repression^19^. By integrating chromatin immunoprecipitation with sequencing (ChIP–seq) data^20,21^, we demonstrate that common variants associated with ANM are enriched in the binding sites of *ZNF518A* (Supplementary Table 7 and Supplementary Information), providing further support for the role of this gene in ovarian ageing.

In addition, there were two genes within 500 kb of GWAS loci (*BRCA1* and *SLCO4A1*) that were associated with ANM by gene burden testing at *P* < 1.7 × 10^−5^. Effect sizes for common variant associations ranged from 0.07–0.24 years per allele at these loci, whereas gene burden tests for rarer variants at these same loci revealed much larger effect sizes: for *BRCA1*, 2.1 years earlier ANM for PTVs (95% CI: 1.2–3.0, *P* = 2.4 × 10^−^^6^) and for *SLCO4A1*, 1.13 years earlier ANM for damaging variants (95% CI: 0.6–1.64, *P* = 1.1 × 10^−5^), with non-overlapping 95% CI between common and rare variant associations for *BRCA1*.

## Non-reproductive health and disease effects

**Extended Data Fig. 3:**
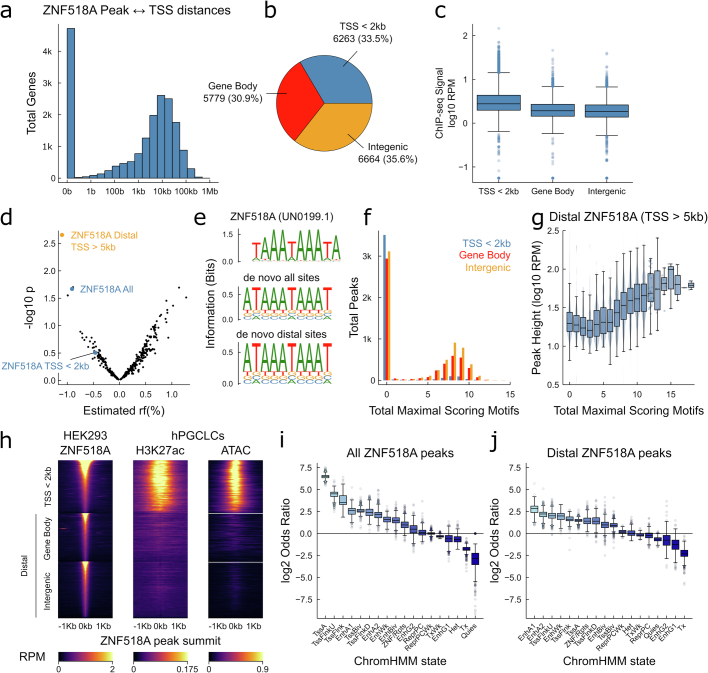
Functional analysis of ZNF518A bound loci. **(a)** Histogram of log10-scale distances between *ZNF518A* and nearest gene transcription start site (TSS). **(b)** Proportion of *ZNF18A* peaks falling proximal to TSS (TSS < 2 kb), within gene bodies and in intergenic regions. **(c)** Boxplots showing total normalised reads per million (RPM) for every peak for categories TSS < 2 kb, gene body and intergenic - *ZNF518A* peaks have greater signal at proximal to TSS. **(d)** SLDP association between ANM GWAS variants and *ZNF518A* peaks, stratified by all peaks, proximal (<2 kb) from a TSS, and distal (>5 kb) from a TSS. The association between ANM variants and *ZNF518A* peaks appears due to distal *ZNF518A* peaks (either gene body or intergenic, >5 kb TSS) and not proximal TSS binding. Numerical results are reported in Supplementary Table 7. **(e)** De novo motif discovery recovers unvalidated JASPAR motif for *ZNF518A* UN0199.1. Homer enrichment statistics: all sites P = 10^−6451^ motif in 31.2% of targets (1.15% background); distal sites P = 10^−4590^ motif in 47.3% of targets (1.81 % background). **(f)** Proportion of maximal scoring instances of UN0199.1 (sequences that exactly match motif consensus) by *ZNF518A* peak category. Many distal peaks contain multiple perfect instances of the motif. **(g)** Boxplots, violin plots and dot plots depicting the relationship between *ZNF518A* ChIP-seq peak height and number of maximal scoring motifs present in peak. A strong relationship between peak height and number of motif instances can be observed. **(h)** Heatmaps depicting *ZNF518A* ChIP-seq, H3K27ac ChIP-seq in hPGCLCs, and chromatin accessibility by ATAC-seq in hPGCLCs. Signal shown over all *ZNF518A* peaks in RPM + /− 1 kb of *ZNF518A* peak summit. *ZNF518A* bound promoters (TSS < 2 kb) are accessible and are marked with H3K27ac, distal regions either in gene bodies or intergenic regions show no H3K27ac or chromatin accessibility, suggestive that *ZNF518A* represses these regulatory regions. **(i,j)** Association shown in odds ratios of ChromHMM states over 833 tissues/cell types from Epimap; boxplots with outliers shown, with each boxplot summarising the distribution of associations over all tissues/cell types for a given chromatin state. **(i)** All *ZNF518A* peaks; **(j)** *ZNF518A* peaks distal from TSS.

Our genetic studies have previously shown that the genetic mechanisms that regulate the end of reproductive life are largely distinct from those that determine its beginning^22,23^. However, it is noteworthy that the largest reported GWAS for age at menarche identified a common variant signal at the *ZNF518A* locus for later puberty timing in girls (rs1172955, beta = 0.04 years (95% CI: 0.03–0.05), *P* = 6.6 × 10^−12^), which appears nominally associated with earlier ANM^22^ (beta = −0.04 yeaers (95% CI: 0.01–0.06), *P* = 6.6 × 10^−3^). To extend this observation, we found that our identified *ZNF518A* PTVs were also associated with later age at menarche (0.56 years (95% CI: 0.14–0.98), *P* = 9.2 × 10^−3^). Furthermore, using functional genome-wide association analysis^24^ and signed linkage disequilibrium profile^25^ (SLDP), we found that similar to ANM, common variants that associate with puberty in girls were enriched in transcriptional targets of *ZNF518A* (Extended Data Fig. 3 and Supplementary Table 7). These data suggest that loss of *ZNF518A* shortens reproductive lifespan by delaying puberty and reducing age at menopause.

**Fig. 3:**
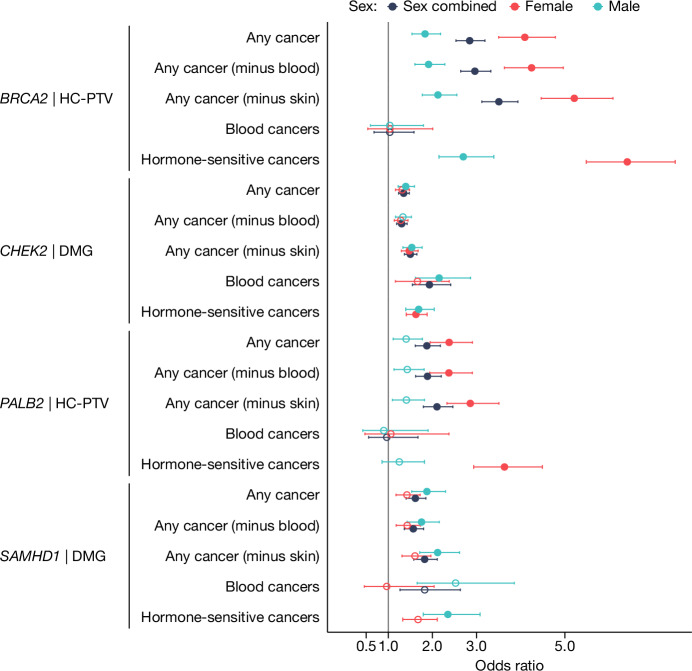
Forest plot for ANM WES genes with significant gene burden associations for cancer phenotypes. Exome-wide significant (*P* < 1.08 × 10^−6^) genes are displayed, showing sex-stratified and combined results from BOLT-LMM analysis. Hormone-sensitive cancers only were tested in male and female subjects separately (Methods). The presented masks were selected on the basis of the most significant association per gene and cancer type. Points and bars indicate odds ratio and 95% CI, respectively, for specific genes and their variant categories for each cancer type (values are given in Supplementary Table 10). A filled circle indicates that a result passes a Bonferroni-corrected significance threshold of *P* < 1.08 × 10^−6^; an unfilled circle indicates a nonsignificant association. *n* = 421,064 (228,517 female and 192,547 male participants).

We next explored the effect of ANM-associated genes on cancer outcomes, replicating previously reported associations with PTVs in *BRCA2*, *CHEK2* and *PALB2* and cancer outcomes in male and female subjects^1,10^ (Supplementary Tables 8–10). We also identified a novel association of *SAMHD1* damaging variants and HC-PTVs with ‘all cancer’ in both males (odds ratio (OR) = 2.12 (95% CI: 1.72–2.62), *P* = 4.7 × 10^−13^) and females (OR = 1.61 (95% CI: 1.31–1.96), *P* = 4 × 10^−6^; Fig. 3 and Supplementary Tables 8–10).

**Fig. 4:**
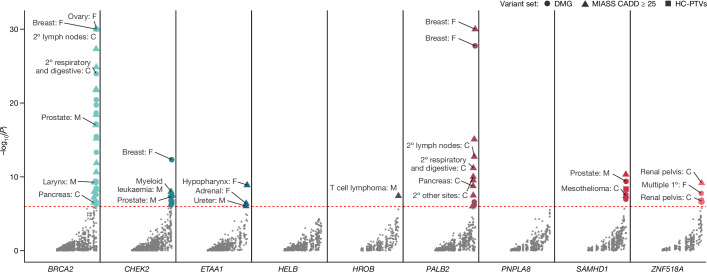
Genetic susceptibility to premature ovarian ageing and increased risk for diverse cancer types. Association between loss of ANM genes identified in this study and risk of 90 site-specific cancers among UK Biobank participants. Summary statistics for cancer associations were obtained using a logistic regression with penalized likelihood estimation that controls for case–control imbalance^33^ (Methods). Associations highlighted with text labels passed an exome-wide significance threshold (*P* < 1.08 × 10^−6^). The *y* axis is capped at −log_10_(P) = 30 for visualization purposes; uncapped summary statistics are presented in Supplementary Table 11. 1°, primary cancer; 2°, secondary cancer; F, female participants; M, male participants; C, both sexes combined.

*SAMHD1* associations with cancer appear to be driven by increased risk for multiple site-specific cancers, notably prostate cancer in men, mesothelioma in both men and women, and suggestive evidence for higher breast cancer susceptibility in women (Fig. 4 and Supplementary Table 11). Although the numbers of mutation carriers diagnosed with each site-specific cancer was small, the majority of these findings persisted using logistic regression with penalized likelihood estimation, which is more robust to extreme case–control imbalance^26^ (Supplementary Table 11). To replicate this association, we interrogated genetic data in up to 49,981 cancer cases and 337,946 controls from the Icelandic deCODE study^16,17^. We observed highly similar results (Supplementary Table 12) to those from the UK Biobank, demonstrating increased all-site cancer susceptibility in male (OR = 1.67 (95% CI: 1.18–2.37), *P* = 0.004), female (OR = 1.57 (95% CI: 1.15–2.15), *P* = 0.005) and sex-combined models (OR = 1.61 (95% CI: 1.28–2.03), *P* = 6 × 10^−5^). Significant associations were also seen for a number of site-specific cancers, including haematological cancers in men (OR = 4.18 (95% CI: 1.90–9.21), *P* = 3.9 × 10^−4^) and prostate cancer (OR = 2.36 (95% CI: 1.14–4.87), *P* = 0.02).

**Extended Data Fig. 4:**
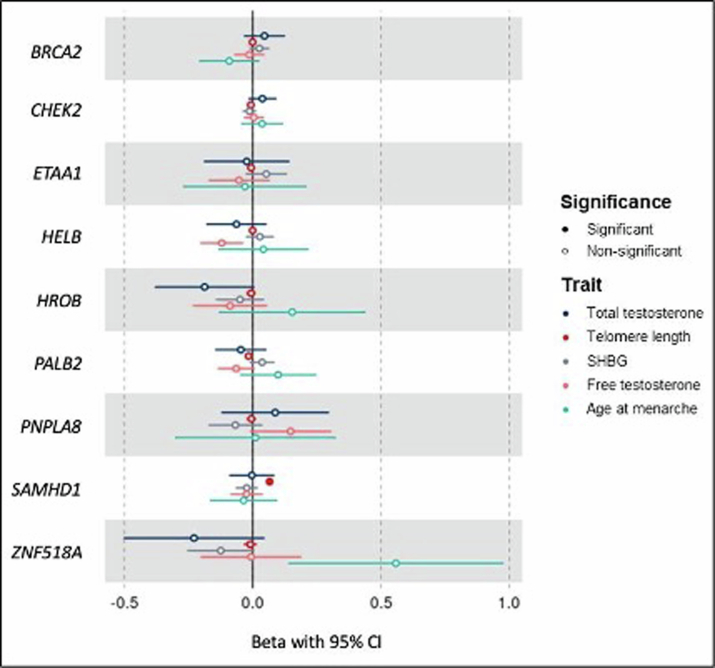
ANM gene burden associations with reproductive ageing-related traits of interest in females only. The coefficients and 95% CIs were female-specific and plotted for the quantitative traits only. The association was tested using BOLT-LMM. Male-specific and sex combined associations could be found in Supplementary Table 10.

Cancer risk-increasing alleles in *SAMHD1* were associated with later ANM, following a similar pattern demonstrated previously for *CHEK2*. This finding is consistent with a mechanism of disrupted DNA damage sensing and apoptosis, resulting in slowed depletion of the ovarian reserve^1^. We note, however, that there are other mechanisms of ovarian reserve depletion, and future experimental work should seek to better understand this specific association. In addition, we provide robust evidence for a previously described rare variant association for *SAMHD1* with telomere length^27^, highlighting that rare damaging variants cause longer telomere length (*P* = 1.4 × 10^−59^) (Extended Data Fig. 4 and Supplementary Table 10).

## Effects on de novo mutation rate

Of the nine genes that we identified in our exome analysis as associated with ANM, seven are involved in DNA damage repair, further supporting the role of these pathways in ovarian ageing (Supplementary Table 13). For genes that inhibit DNA double-strand break repair, the hypothesis is that they cause premature depletion of the ovarian reserve owing to a failure to repair oocytes with DNA damage^1^. This is evidenced by the reported increased numbers of DNA double strand breaks in the oocytes of *Brca1*-deficient mice and of women with *BRCA1* mutations who underwent elective oophorectomy^28–30^. Our current study adds further support for this hypothesis, with heterozygous *BRCA1* and *BRCA2* loss-of-function alleles being associated with 2.1 and 1.18 years earlier ANM, respectively.

**Extended Data Fig. 5:**
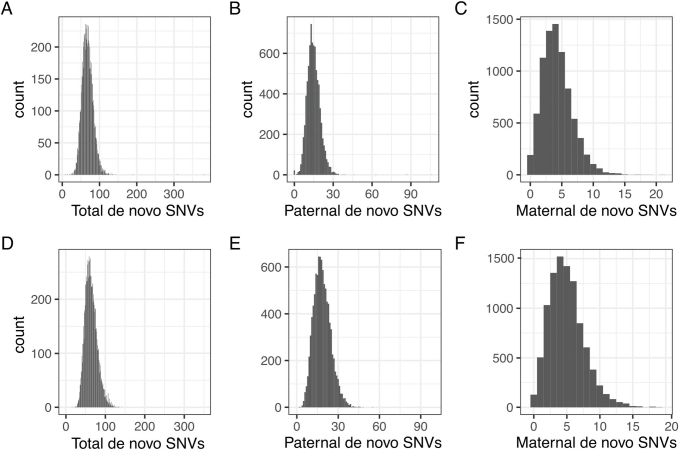
Distribution of de novo single nucleotide variants (dnSNVs). The histogram shows the number of **(A)** total dnSNVs, includes both phased (maternal + paternal) and unphased DNMs, **(B)** paternally derived dnSNVs and **(C)** maternally derived dnSNVs in unrelated probands with European ancestry from the 100,000 Genomes Project. **(D–F)** show similar distributions in individuals from deCODE.

We sought to build on these observations by testing the hypothesis that inter-individual variation in these DDR processes would influence the mutation rate in germ cells and thus in the offspring. More specifically, we hypothesized that genetic susceptibility to earlier ovarian ageing would be associated with a higher de novo mutation (DNM) rate in offspring. To test this, we analysed whole-genome-sequenced parent–offspring trios from the 100,000 Genome Project (100kGP)^31^ (*n* = 8,809 with European ancestry) and followed up in trios from the deCODE study^32,33^ (*n* = 6,042) (Extended Data Fig. 5). We calculated a polygenic score (PGS) for ANM in the parents by combining the effect estimates from our previously identified 290 common variants^1^ and tested this for association with the phased DNM rate in the offspring, adjusting for parental age and quality control-related covariates. In the 100kGP dataset, we found that maternal genetic susceptibility to earlier ANM was associated with an increased rate of maternally derived DNMs in the offspring (Poisson regression; meta-analysis beta = −0.082 DNMs per s.d. increase in the PGS (95% CI: −0.126, −0.037), *P* = 0.00033; Supplementary Table 14). However, this association was not replicated in the deCODE dataset (beta = 0.018 (95% CI: −0.038, 0.073), *P* = 0.53), and the estimates from the 100kGP and deCODE data were inconsistent (heterogeneity *P* = 0.006). A meta-analysis of the results across the two cohorts gave a significant effect of maternal ANM PGS on maternally derived DNMs (beta = −0.0426 (95% CI: −0.0772, −0.0079) DNMs per s.d. increase in the PGS; standard error = 0.018, *P* = 0.016). The 100kGP finding was consistent in sensitivity analyses using a two-sample Mendelian randomization framework that can better model the dose–response relationship of these variants (Supplementary Table 15). These 100kGP Mendelian randomization results were highly concordant, with all models showing a significant result (minumum *P* value = 6.3 × 10^−5^) and no heterogeneity in effect for ANM genetic variants used as instrumental variables (Methods). In both 100kGP and deCODE data, the paternal PGS was not associated with paternally or maternally derived DNMs (*P* > 0.05), or the maternal PGS associated with paternally derived DNMs (*P* > 0.05). Finally, we tested whether rare damaging variants in the nine ANM-associated genes (Fig. 2) were associated with DNM rate in the 100kGP and deCODE study (Supplementary Table 16). After meta-analysis and following multiple testing correction (*P* > 0.05/(2 × 9)), none of the nine genes showed significant rare variant associations with DNM rate in either mothers or fathers.

## Discussion

Our study extends the number of genes implicated in ovarian ageing through the identification of rare protein-coding variants. Effect sizes ranged from 5.61 years earlier ANM for HC-PTV carriers in *ZNF518A*, to 1.35 years later ANM for women carrying damaging variants in *SAMHD1* compared with a maximum effect size of 1.06 years (median 0.12 years) reported for common variants^1^ (MAF > 1%). Several of these effect estimates were comparable to those conferred by *FMR1* premutations, which are currently used as part of the only routinely applied clinical genetics test for premature ovarian insufficiency^34^. Deleterious variants in three genes (*CHEK2*, *HELB* and *SAMHD1*) were associated with an increase in ANM and therefore represent potential therapeutic targets for enhancing ovarian stimulation in women undergoing in vitro fertilization treatment through short-term apoptotic inhibition. Seven out of the nine ANM genes identified have known roles in DNA damage repair, and—to our knowledge—three of these are linked to ANM for the first time (*PALB2*, *ETAA1* and *HROB*). PALB2 is involved in BRCA2 localization and stability, and *PALB2* compound heterozygous mutations result in Fanconi anaemia and predispose to childhood malignancies^35^. ETAA1 accumulates at DNA damage sites in response to replication stress^36,37^, and HROB is involved in homologous recombination by recruiting the MCM8–MCM9 helicase to sites of DNA damage to promote DNA synthesis^38,39^. Homozygous loss of function of *HROB* is associated with premature ovarian insufficiency^40^ and infertility in both sexes in mouse models^38^.

Novel biological mechanisms of ovarian ageing were revealed by finding associations with two non-DDR genes (*PNPLA8* and *ZNF518A*): PNPLA8 is a calcium-independent phospholipase^41,42^ and a recessive cause of neurodegenerative mitochondrial disease and mitochondrial myopathy^43–45^; to our knowledge, an association with reproductive phenotypes has not been described previously. *ZNF518A* belongs to the zinc-finger protein family and is likely to be a transcriptional regulator for a large number of genes^19^. We found that female carriers of rare PTVs in *ZNF518A* have shorter reproductive lifespan owing to delayed puberty timing and earlier menopause. Enrichment of GWAS signals at *ZNF518A* binding sites suggests that *ZNF518A* regulates the genes involved in reproductive longevity by repression of regulatory elements distal to their transcription start sites. *ZNF518A* has also recently been demonstrated to have a role in forming heterochromatin at pericentromeric regions, which is essential for proper chromosome segregation during mitosis and meiosis^46^.

Whereas mutation in *SAMHD1* is a common somatic event in a variety of cancers^47^, we demonstrate here that it is also a germline risk factor. Recessive inheritance of *SAMHD1* missense variants and PTVs have been associated with Aicardi–Goutières syndrome, a congenital autoimmune disease^48^. The damaging variants in *SAMHD1* that we identified are associated with increased risk of ‘all cancer’ in men and women, as well as in sex-specific cancers, highlighting *SAMHD1* as a novel risk factor for prostate cancer in men and hormone-sensitive cancers in women. Recent studies have demonstrated association of germline *SAMHD1* coding variants with having two or more primary cancers in the UK Biobank^49^ (*P* = 2.4 × 10^−7^), and with breast cancer susceptibility^50^ (*P* < 1 × 10^−4^). *SAMHD1* has a role in preventing the accumulation of excess deoxynucleotide triphosphates (dNTPs), particularly in non-dividing cells^51^. A regulated dNTP pool is important for the fidelity of DNA repair, thus highlighting additional roles of this gene in facilitation of DNA end resection during DNA replication and repair^51–56^. *SAMHD1* deficiency leads to resistance to apoptosis^57,58^, suggesting that delayed ANM might originate from slowed depletion of ovarian reserve due to disrupted apoptosis, analogous to the mechanism for *CHEK2* that has been reported previously.

Previous studies have demonstrated that parental age is strongly associated with the number of de novo mutations in offspring^59^, with the majority of these mutations arising from the high rate of spermatogonial stem cell divisions that underlie spermatogenesis throughout the adult life of men^60^. We investigated whether women at higher genetic risk of earlier menopause transmit more de novo mutations to offspring. Whereas we found significant evidence for this in 100kGP data, we could not replicate the association between ANM PGS and maternally derived DNMs in deCODE data. We cannot currently explain this finding, other than with the possibility that our result from 100kGP is inflated by ‘winner’s curse’ and that the true effect size is lower than our point estimate. Power calculations suggest that the deCODE dataset is well-powered to replicate the effect if it is equal to our point estimate in 100kGP (>90% power) but has only modest power (around 40%) if the effect is at the lower bound of our 95% CI from 100kGP. Future large studies of whole-genome sequence data in trios will be important to further explore this relationship. If confirmed, this finding could have direct implications for the health of future generations, given the widely reported link between de novo mutations and increased risk of psychiatric disease and developmental disorders^61–64^. If genetic susceptibility to earlier menopause influences de novo mutation rate, non-genetic risk factors for earlier ANM, such as smoking and alcohol intake, would probably have the same effect, and there is some evidence to support this^65^. Our observations make conceptual sense given that menopause timing appears to be primarily driven by the genetic integrity of oocytes and their ability to sustain, detect, repair and respond to acquired DNA damage^1^. These observations also build on earlier work in mice and humans that *BRCA1*/*2* deficiency increases the rate of double strand breaks in oocytes and reduces ovarian reserve^28–30^.

A limitation of our work is that a small proportion of maternally phased DNMs could be postzygotic mutations in the child, which did not originate in the maternal germline. We were unable to differentiate between these owing to the modest sequencing coverage of a single tissue per child. A further limitation is that, owing to data availability, analyses have been restricted to women of European ancestry, making it difficult to evaluate how generalizable these findings may be to other populations, as average age of menopause in women from different ancestry groups varies^66^. We anticipate that this will be addressed in future studies as relevant data become available.

Our study of rare coding variation across the genome expands our understanding of the genetic architecture of ovarian ageing. Future genomic studies incorporating rare non-coding variation in addition to experimental work will build on our identified genetic associations to help further our understanding of the underlying biological mechanisms governing ovarian ageing.

## Methods

### UK Biobank data processing and quality control

To conduct the rare variant burden analyses described in this study, we obtained WES data for 454,787 individuals from the UK Biobank study^67^. Participants were excluded on the basis of excess heterozygosity, autosomal variant missingness on genotyping arrays (≥5%), or inclusion in the subset of phased samples as defined in Bycroft et al.^68^. Analysis was restricted to participants with European genetic ancestry, owing to the unknown influence of rare variants on population stratification and limited non-European sample size, leaving a total of 421,065 individuals. Variant quality control and annotation were performed using the UK Biobank Research Analysis Platform (RAP; https://ukbiobank.dnanexus.com/), a cloud-based central data repository for UK Biobank WES and phenotypic data. Besides the quality control described by Backman et al.^67^, we performed additional steps using custom applets designed for the RAP. First, we processed population-level variant call format (VCF) files by splitting and left-correcting multi-allelic variants into separate alleles using ‘bcftools norm’^69^. Second, we performed genotype-level filtering applying ‘bcftools filter’ separately for single nucleotide variants (SNVs) and insertions–deletion mutations using a missingness-based approach. Using this approach, we set to missing (./.) all SNV genotypes with depth <7 and genotype quality <20 or insertion–deletion genotypes with a depth <10 and genotype quality <20. Next, we applied a binomial test to assess an expected alternate allele contribution of 50% for heterozygous SNVs; we set to missing all SNV genotypes with a binomial test *P* value ≤ 1 × 10^−3^. Following genotype-level filtering we recalculated the proportion of individuals with a missing genotype for each variant and filtered all variants with a missingness value > 50%. The variant annotation was performed using the ENSEMBL variant effect predictor (VEP) v104^70^ with the ‘--everything’ flag and plugins for CADD^71^ and LOFTEE^72^ enabled. For each variant we prioritized the highest impact individual consequence as defined by VEP and one ENSEMBL transcript as determined by whether or not the annotated transcript was protein-coding, MANE select v0.97, or the VEP canonical transcript. Following annotation, variants were categorised on the basis of their predicted impact on the annotated transcript. PTVs were defined as all variants annotated as stop-gained, frameshift, splice acceptor and splice donor. Missense variant consequences are identical to those defined by VEP. Only autosomal or chromosome X variants within ENSEMBL protein-coding transcripts and within transcripts included on the UK Biobank ES assay^67^ were retained for subsequent burden testing.

### Exome-wide association analyses in the UK Biobank

To perform rare variant burden tests, we used a custom implementation of BOLT-LMM v2.3.6^15^ for the RAP. Two primary inputs are required by BOLT-LMM: (1) a set of genotypes with minor allele count >100 derived from genotyping arrays to construct a null linear mixed effects model; and (2) a larger set of variants collapsed on ENSEMBL transcript to perform association tests. For the former, we queried genotyping data available on the RAP and restricted to an identical set of individuals included for rare variant association tests. For the latter, and as BOLT-LMM expects imputed genotyping data as input rather than per-gene carrier status, we created dummy genotype files where each variant represents one gene and individuals with a qualifying variant within that gene are coded as heterozygous, regardless of the number of variants that individual has in that gene.

To test a range of variant annotation categories for MAF < 0.1%, we created dummy genotype files for high confidence PTVs as defined by LOFTEE, missense variants with CADD ≥ 25, and damaging variants that included both high confidence PTVs and missense variants with CADD ≥ 25. For each phenotype tested, BOLT-LMM was then run with default parameters other than the inclusion of the ‘lmmInfOnly’ flag. To derive association statistics for individual markers, we also provided all 26,657,229 individual markers regardless of filtering status as input to BOLT-LMM. All tested phenotypes were run as continuous traits corrected by age, age^2^, sex, the first ten genetic principal components as calculated in Bycroft et al.^68^ and study participant ES batch as a categorical covariate (50k, 200k or 450k).

For discovery analysis in the primary trait of interest, ANM, we analysed 17,475 protein-coding genes with the minimum of 10 rare allele carriers in at least one of the masks tested using BOLT-LMM (Supplementary Table 1). The significant gene-level associations for ANM were identified applying Bonferroni correction for the number of masks with MAC ≥ 10 (*n* = 46,251 masks) in 17,475 protein-coding genes (*P*: 0.05/46,251 = 1.08 × 10^−6^) (Supplementary Table 2). Furthermore, to compare and explain potential differences between our WES results and the previously published one^5^, we ran the above approach using MAF < 1%, a cut-off applied by Ward et al. (Supplementary Table 5 and Supplementary Information).

To generate accurate odds ratio and standard error estimates for binary traits, we also implemented a generalized linear model using the statsmodels package^73^ for Python in a three-step process. First, a null model was run with the phenotype as a continuous trait, corrected for control covariates as described above. Second, we regressed carrier status for individual genes on the residuals of the null model to obtain a preliminary *P* value. Thirdly, all genes were again tested using a full model to obtain odds ratios and standard errors with the family set to ‘binomial’. Generalized linear models utilized identical input to BOLT-LMM converted to a sparse matrix.

### ANM phenotype derivation

ANM was derived for individuals within the UK Biobank, who were deemed to have undergone natural menopause—that is, not affected by surgical or pharmaceutical interventions, as follows.

First, European female participants (*n* = 245,820) who indicated during any of the attended visits having had a hysterectomy were collated (fields 3591 and 2724) and their reported hysterectomy ages were extracted (field 2824) and the median age was kept (*n* = 47,218 and 46,260 with reported ages). The same procedure was followed for participants indicating having undergone a bilateral oophorectomy (surgery field 2834 and age field 3882, *n* = 20,495 and 20,001 with reported ages).

For individuals having indicated the use of hormone-replacement therapy (HRT; field 2814), HRT start and end ages were collated (fields 3536 and 3546, accordingly) across the different attended visits (*n* = 98,104). In cases where the reported chronological HRT age at later attended visits was greater than that at previous visits, the later instances were prioritised, i.e. as they would potentially indicate an updated use of HRT. In cases where different HRT ages were reported, but not in chronologically increasing order, the median age was kept.

Menopausal status was determined using data across instances (field 2724) and prioritizing the latest reported data, to account for changes in menopause status. For participants indicating having undergone menopause, their reported ages at menopause were collated (field 3581) using the same procedure as for HRT ages (*n* = 158,264).

Exclusions were then applied to this age at menopause, as follows:

- Participants reporting undergoing a hysterectomy and/or oophorectomy, but not the age at which this happened (*n* = 958 and 494, accordingly).
- Participants reporting multiple hysterectomy and/or oophorectomy ages, which were more than 10 years apart (*n* = 38 and 23, accordingly).
- Participants reporting multiple HRT start and/or end ages, which were not in chronologically ascending order and were more than 10 years apart (*n* = 124 and 137, accordingly).
- Participants reporting multiple ages at menopause, which were not in chronologically ascending order and were more than 10 years apart (*n* = 73) and participants who reported both having and not having been through menopause and no other interventions (*n* = 98).
- Participants having undergone a hysterectomy or oophorectomy before or during the year they report undergoing menopause.
- Participants starting HRT prior to undergoing menopause and participants reporting HRT use, with no accompanying dates.

The resulting trait was representative of an ANM (*n* = 115,051) and was used in downstream analyses. Two additional ANM traits were also calculated, winsorized one by coding everyone reporting an ANM younger than 34, as 34 used in the discovery analysis as the primary phenotype (*n* = 115,051 total, reduced to 106,973 after covariate-resulting exclusions), and one by only including participants reporting ANM between 40 and 60, inclusive (*n* = 104,506), treated as a sensitivity analysis.

All manipulations were conducted in R (v4.1.2) on the UK Biobank RAP (https://ukbiobank.dnanexus.com/).

### Replication of rare variant associations

Replication was performed using two study populations: the Icelandic deCODE study^16,17^ and the BRIDGES study^18^.

#### deCODE

The burden test associations are shown for three categories of rare variants with MAF < 2%; (1) loss-of-function (LOF) variants; (2) combination of LOF variants and predicted deleterious missense variants; and (3) combination of LOF variants and missense variants with CADD score ≥ 25. We furthermore show results for category 3 using a more stringent frequency threshold, 0.1%. We included missense variants predicted to cause LOF by two meta-predictors, MetaSVM and MetaLR^74^, using variants available in dbNSFP v4.1c^75^. We used VEP^70^ to attribute predicted consequences to the variants sequenced. For case–control analyses, we used logistic regression and an additive model to test for association between LOF gene burdens and phenotypes, in which disease status was the dependent variable and genotype counts as the independent variable. Individuals were coded 1 if they carry any predicted LOF in the autosomal gene being tested and 0 otherwise. Age, sex and sequencing status of individuals was used as a covariate in the associations. For the analyses, we used software developed at deCODE genetics and we used linkage disequilibrium (LD) score regression intercepts^76^ to adjust the *χ*^2^ statistics and avoid inflation due to cryptic relatedness and stratification. Quantitative traits were analysed using a linear mixed model implemented in BOLT-LMM^15^. To estimate the quality of the sequence variants across the entire set we regressed the alternative allele counts (AD) on the depth (DP) conditioned on the genotypes (GT) reported by GraphTyper^77^. For a well-behaving sequence variant, the mean alternative allele count for a homozygous reference genotype should be 0, for a heterozygous genotype it should be DP/2 and for homozygous alternative genotype it should be DP. Under the assumption of no sequencing or genotyping error, the expected value of AD should be DP conditioned on the genotype, in other words an identity line (slope 1 and intercept 0). Deviations from the identity line indicate that the sequence variant is spurious or somatic. We filter variants with slope less than 0.5. Additionally, GraphTyper employs a logistic regression model that assigns each variant a score (AAscore) predicting the probability that it is a true positive. We used only variants that have a AAscore > 0.8.

#### BRIDGES

The BRIDGES study included women from studies participating in the Breast Cancer Association Consortium (BCAC; v14) (http://bcac.ccge.medschl.cam.ac.uk/). The subset of population or hospital-based studies sampled independently of family history, together with population-matched controls (25 studies) were included in the analyses. ANM (years) was obtained from baseline questionnaire data. Women were considered as having experienced natural menopause if they indicated that the reason for menopause was reported as ‘natural’ or ‘unknown’. Women were excluded from the analysis if the reason was indicated as either oophorectomy, hysterectomy, chemotherapy, stopping oral contraception or ‘any other reason’. Only studies with information on year of birth and age at menopause, and only women with reported age at menopause between ages twenty-five years and sixty years were included. All studies were approved by the relevant ethical review boards and used appropriate consent procedures.

Targeted sequencing of germline DNA from participants for 35 known or suspected breast cancer genes was performed, including the coding sequence and splice sites. Details of library preparation, sequencing, variant calling, and quality control procedures are described in Dorling et al.^18^. Carriers of PTVs in more than one of five main breast cancer susceptibility genes (*BRCA1*, *BRCA2*, *ATM*, *CHEK2*, *PALB2*) were excluded. Carriers of pathogenic missense variants (as defined by Dorling et al.^18^) in *BRCA1* or *BRCA2* were also excluded.

We carried out burden analyses, assessing the associations between rare variants in aggregate and ANM using linear regression, adjusting for country of origin, breast cancer case-control status and year of birth (categorized as up to 1935, 1936–1945, 1946–1955 or after 1956), and for some analyses body mass index (BMI). For each gene we considered PTVs in aggregate. The primary analyses included covariates to adjust for population, which was defined by country, with the exception of Malaysia and Singapore, in which the three distinct ethnic groups (Chinese, Indian and Malay) were treated as different strata and the UK, which was treated as separate strata (SEARCH, from East Anglia and PROCAS from north-west England). Sensitivity analyses were carried out adjusting for BMI in women with recorded age at BMI, and among women without a diagnosis of breast cancer. Sensitivity analyses were also carried out defining non-carriers as women not harbouring PTVs in the five main genes or pathogenic MSVs in *BRCA1* and *BRCA2*.

### WES sensitivity analysis using REGENIE

To replicate the primary findings and account for potential bias that could be introduced by exclusively using one discovery approach, a second analyst independently derived the age at menopause phenotype using a previously published method^78^ and conducted additional burden association analysis using the REGENIE regression algorithm (REGENIEv2.2.4; https://github.com/rgcgithub/regenie). REGENIE implements a generalized mixed-model region-based association test that can account for population stratification and sample relatedness in large-scale analyses. REGENIE runs in two steps^79^, which we implemented on the UK Biobank RAP. In the first step, genetic variants are aggregated into gene-specific units for each class of variant, called masks. We selected variants in CCDS transcripts deemed to be high confidence by LOFTEE^72^ with MAF < 0.1% and annotated using VEP^70^. We created three masks, independently of the primary analysis group: (1) LOF variants (stop-gain, frameshift, or abolishing a canonical splice site (−2 or +2 bp from exon, excluding the ones in the last exon)) or missense variants with CADD score >30; (2) LOF or missense variants with CADD score >25; and (3) all missense variants. In the second step, the three masks were tested for association with ANM. We applied an inverse normal rank transformation to ANM and included recruitment centre, sequence batch and 40 principal components as covariates. For each gene, we present results for the transcript with the smallest burden *P* value. We performed a sensitivity analysis, excluding women who had any cancer diagnosis before ANM (ICD10 C00-C97 excluding C44, ICD9 140-208 excluding 173; *n* = 2,585). The results for the sensitivity analyses performed via REGENIE are available in Supplementary Tables 1 and 2.

### Common variant GWAS lookups

Genes within 500 kb upstream and downstream of the 290 lead SNPs from the latest GWAS of ANM^1^ were extracted from the exome-wide analysis. There were a total of 2149 genes within the GWAS regions. Burden tests in these genes with a Bonferroni corrected *P* value of <2.3 × 10^−5^ (0.05/2,149) were highlighted. The results are available in Supplementary Table 6.

### Phenome-wide association analysis

To test the association of ANM identified genes in other phenotypes, we processed additional reproductive ageing-related phenotypes, including age at menarche, cancer, telomere length and sex hormones. All tested phenotypes were run as either continuous (age at menarche, telomere length and sex hormones) or binary traits (cancer) corrected by age, age^2^, sex, the first ten genetic principal components as calculated in Bycroft et al.^68^, and study participant ES batch as a categorical covariate (either 50k, 200k or 450k). Phenotype definitions and processing used in this study are described in Supplementary Tables 8 and 9. Only the first instance (initial visit) was used for generating all phenotype definitions unless specifically noted in Supplementary Table 8. In case of cancer-specific analysis, data from cancer registries, death records, hospital admissions and self-reported were harmonized to ICD10 coding. If a participant had a code for any of the cancers recorded in ICD10 (C00-C97) then they were counted as a case for this phenotype. Minimal filtering was performed on the data, with only those cases where a diagnosis of sex-specific cancer was given in contrast to the sex data contained in UK Biobank record 31, was a diagnosis not used. For more information on cancer-specific analysis refer to Supplementary Tables 9 and 11.

### Cancer PheWAS associations

To test for an association between genes we identified as associated with menopause timing (Fig. 2 and Supplementary Table 2) and 90 individual cancers as included in cancer registries, death records, hospital admissions and self-reported data provided by UK Biobank (for example, breast, prostate, etc.) we utilised a logistic model with identical covariates as used during gene burden testing (*n* = 2430 tests) (Supplementary Tables 9 and 11). As standard logistic regression can lead to inflated test statistic estimates in cases of severe case/control imbalance^80^, we also performed a logistic regression with penalised likelihood estimation as described by Firth^26^ (Supplementary Table 11). Models were run as discussed in Kosmidis et al.^81^ using the brglm2 package implemented in R. brglm2 was run via the glm function with default parameters other than “family” set to “binomial”, method set to “brglmFit”, and type set to “AS_mean”.

### Expression in human female germ cells

**Extended Data Fig. 6:**
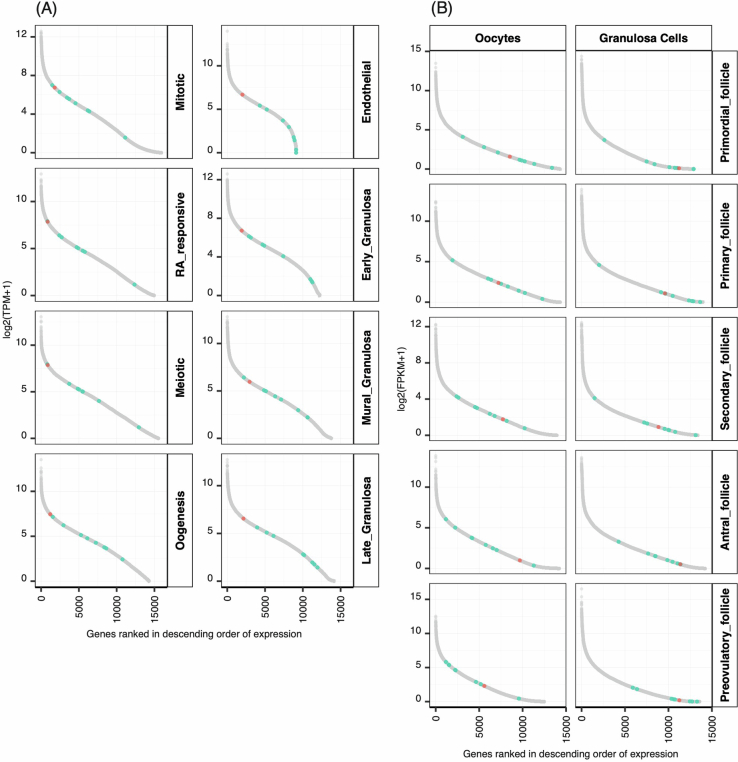
Expression levels of genes across various stages of female germ cell development. In the X-axis, genes are ranked according to their average expression at each stage (Y-axis) **(A)** in human foetal primordial germ cells and **(B)** in granulosa cells in adult follicles. Genes identified as novel ANM genes in WES analysis are coloured in green and all other genes in the genome are in grey. *ZNF518A* is depicted in orange for the ease of comparison with other genes (Supplementary Table 17).

**Extended Data Fig. 7:**
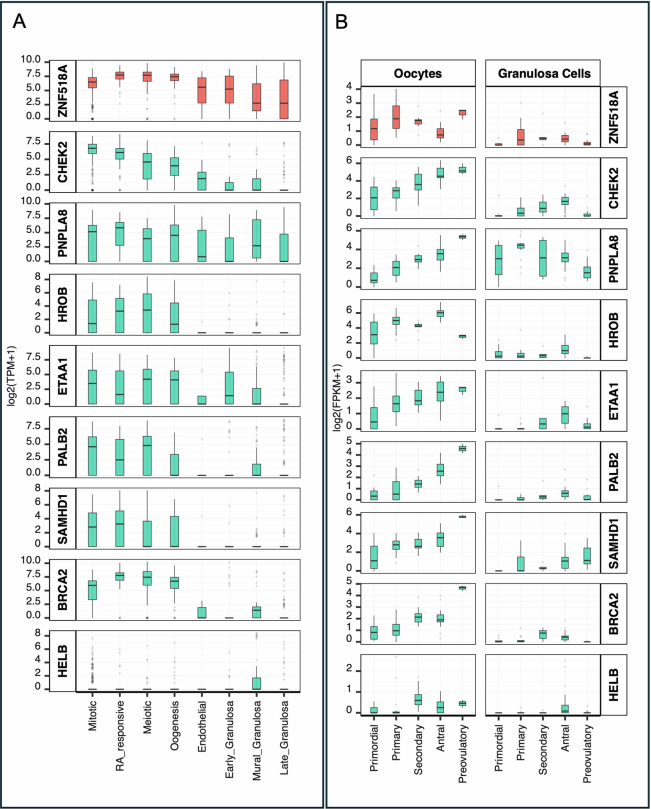
mRNA expression of WES genes during foetal stages and folliculogenesis. Box and whisker plots of mRNA expression of the WES genes at different stages of germ cell development. The plots represent the interquartile range of TPM values, the line at the centre of the box represents the median, error bars indicate the 95% CI and outliers are shown as dots. **(A)** Sub-clusters from single foetal cells from week 5 to 26 post-fertilisation are on the X-axis with the average TPM expression values log2(TPM + 1) on the Y-axis. **(B)** Different stages of folliculogenesis in oocytes and granulosa cells are represented on the X-axis with their average expression values log2(FPKM + 1) on the Y-axis (Supplementary Table 18).

We studied the mRNA abundance of WES genes during various stages of human female germ cell development using single-cell RNA sequencing data. We used the processed single cell RNA resequencing datasets from two published studies (Extended Data Figs. 6 and 7 and Supplementary Tables 17 and 18). This included single-cell RNA sequencing data from fetal primordial germ cells of human female embryos (accession code: GSE86146^82^), and from oocyte and granulosa cell fractions during various stages of follicle development (accession code: GSE107746^83^). A pseudo score of 1 was added to all values before log transformation of the dataset. The samples from fetal germ cells were categorized into sub-clusters as defined in the original study. The study by Li et al.^82^ identified 17 clusters by performing a *t*-distributed stochastic neighbour embedding analysis and using expression profiles of known marker genes for various stages of fetal germ cell development. In our analysis we have included four clusters of female fetal germ cells (mitotic, retinoic acid-responsive, meiotic and oogenesis) and four clusters containing somatic cells in the fetal gonads (endothelial, early_granulosa, mural_granulosa and late_granulosa). Software packages for R—tidyverse (https://www.tidyverse.org/), pheatmap, (https://CRAN.R-project.org/package=pheatmap) and reshape2 (https://github.com/hadley/reshape)—were used in processing and visualising the data.

### De novo mutation rate analyses in 100kGP

#### Constructing PGSs

We calculated PGSs in participants from the rare disease programme of the 100kGP v14. There are 77,901 individuals in the aggregated variant calls (aggV2) after excluding participants whose genetically inferred sex is not consistent with their phenotypic sex. We restricted the PGS analysis to individuals of European ancestry, which was predicted by the Genomics England bioinformatics team using a random forest model based on genetic principal components generated by projecting aggV2 data onto the 1000 Genomes phase 3 principal component loadings. We removed one sample in each pair of related probands with kinship coefficient > 1/(2^4.5^)—that is, up to and including third-degree relationships. Probands with the highest number of relatives were removed first. Similarly, we retained unrelated mothers and fathers of these unrelated probands. It left us with 8,089 mother–offspring duos and 8,029 father–offspring duos.

We used the lead variants (or proxies, as described below) for genome-wide significant loci previously reported for ANM^1^ to calculate PGS in the parents. In 100kGP, we removed variants with MAF < 0.5% or missing rate >5% from the aggV2 variants prepared by the Genomics England bioinformatics team. For lead variants that did not exist in 100kGP, we used the most significant proxy variants with LD *r*^2^ > 0.5 if available in 100kGP. This resulted in a PGS constructed from 287 of the 290 previously reported loci. We regressed out 20 genetic principal components that were calculated within the European subset from the PGS and scaled the residuals to have mean = 0 and s.d. = 1. Higher PGS indicates later ANM.

#### Calling de novo mutations

De novo mutations (DNMs) were called using the Platypus variant caller in 10,478 parent offspring trios by the Genomics England Bioinformatics team. The detailed analysis pipeline is documented at: https://research-help.genomicsengland.co.uk/display/GERE/De+novo+variant+research+dataset. Extensive quality control and filtering were applied as described previously^31^. In brief, multiple filters were applied, including the following:

- the child had a heterozygous genotype and parents were homozygous reference.
- the parents had <2 reads supporting the alternate allele.
- read depth >20 in child and parents.
- variant allele fraction (VAF) >0.3 and <0.7 in the child.
- no DNMs were clustered (within 20 bp).

Autosomal de novo SNVs (dnSNVs) were phased using reads or read pairs that contained both the dnSNV and heterozygous variants located within 500 bp of it. A DNM was phased to a parent when the DNM appeared exclusively on the same haplotype as its nearby heterozygous variant. About one third of the dnSNVs were phased, of which three quarters were paternally phased (Extended Data Fig. 5 and Supplementary Table 14).

#### Associating the ANM PGS with DNMs in 100kGP

In association models, we accounted for parental age, the primary determinant of the number of DNMs, and various data quality metrics as described^31^:

- Mean coverage for the child, mother and father (child_mean_RD, mother_mean_RD, father_mean_RD).
- Proportion of aligned reads for the child, mother and father (child_prop_aligned, mother_prop_aligned, father_prop_aligned).
- Number of SNVs called for child, mother and father (child_SNVs, mother_SNVs, father_SNVs).
- Median variant allele fraction of DNMs called in child (median_VAF).
- Median Bayes factor as output by Platypus for DNMs called in the child. This is a metric of DNM quality (median_BF).

We first tested the association between parental PGSs and total de novo autosomal SNV count in the offspring in a Poisson regression with an identity link:

$$\begin{array}{l}{\rm{dnSNVs}}\_{\rm{total}}={\beta }_{0}+{\beta }_{1}\mathrm{PGS}({\rm{paternal\; or\; maternal}})\\ \,\,+{\beta }_{2}{\rm{paternal}}\_{\rm{a}}{\rm{g}}{\rm{e}}+{\beta }_{3}{\rm{maternal}}\_{\rm{a}}{\rm{g}}{\rm{e}}{+\beta }_{4}{\rm{child}}\_{\rm{m}}{\rm{e}}{\rm{a}}{\rm{n}}\_{\rm{R}}{\rm{D}}\\ \,\,+{\beta }_{5}{\rm{m}}{\rm{o}}{\rm{t}}{\rm{h}}{\rm{e}}{\rm{r}}\_{\rm{m}}{\rm{e}}{\rm{a}}{\rm{n}}\_{\rm{R}}{\rm{D}}+{\beta }_{6}{\rm{f}}{\rm{a}}{\rm{t}}{\rm{h}}{\rm{e}}{\rm{r}}\_{\rm{m}}{\rm{e}}{\rm{a}}{\rm{n}}\_{\rm{R}}{\rm{D}}\\ \,\,+\,{\beta }_{7}{\rm{c}}{\rm{h}}{\rm{i}}{\rm{l}}{\rm{d}}{\rm{\_}}{\rm{p}}{\rm{r}}{\rm{o}}{\rm{p}}{\rm{\_}}{\rm{a}}{\rm{l}}{\rm{i}}{\rm{g}}{\rm{n}}{\rm{e}}{\rm{d}}+{\beta }_{8}{\rm{m}}{\rm{o}}{\rm{t}}{\rm{h}}{\rm{e}}{\rm{r}}{\rm{\_}}{\rm{p}}{\rm{r}}{\rm{o}}{\rm{p}}{\rm{\_}}{\rm{a}}{\rm{l}}{\rm{i}}{\rm{g}}{\rm{n}}{\rm{e}}\\ \,\,+{\beta }_{9}{\rm{f}}{\rm{a}}{\rm{t}}{\rm{h}}{\rm{e}}{\rm{r}}{\rm{\_}}{\rm{p}}{\rm{r}}{\rm{o}}{\rm{p}}{\rm{\_}}{\rm{a}}{\rm{l}}{\rm{i}}{\rm{g}}{\rm{n}}{\rm{e}}{\rm{d}}+{\beta }_{10}{\rm{c}}{\rm{h}}{\rm{i}}{\rm{l}}{\rm{d}}{\rm{\_}}{\rm{s}}{\rm{n}}{\rm{v}}{\rm{s}}\\ \,\,+{\beta }_{11}{\rm{m}}{\rm{o}}{\rm{t}}{\rm{h}}{\rm{e}}{\rm{r}}{\rm{\_}}{\rm{s}}{\rm{n}}{\rm{v}}{\rm{s}}+{\beta }_{12}{\rm{f}}{\rm{a}}{\rm{t}}{\rm{h}}{\rm{e}}{\rm{r}}{\rm{\_}}{\rm{s}}{\rm{n}}{\rm{v}}{\rm{s}}\\ \,\,+{\beta }_{13}{\rm{m}}{\rm{e}}{\rm{d}}{\rm{i}}{\rm{a}}{\rm{m}}{\rm{\_}}{\rm{V}}{\rm{A}}{\rm{F}}+{\beta }_{14}{\rm{m}}{\rm{e}}{\rm{d}}{\rm{i}}{\rm{a}}{\rm{n}}{\rm{\_}}{\rm{B}}{\rm{F}}\end{array}$$

We also fitted Poisson regression models to test the association between the PGS of one of the parents and the dnSNVs in the offspring that were phased to the relevant parent. We supplied 0.5 as the starting value for all coefficients when running the glm() function in R with an identity link. Using a different starting value (for example, 0.2 and 10) did not change the coefficient estimates.

The paternal model included paternal PGS, age and data quality metrics that are related to the proband and the father:

$$\begin{array}{l}{\rm{dnSNVs}}\_{\rm{paternal}}={\beta }_{0}+{\beta }_{1}{\rm{paternal}}\_{\rm{P}}{\rm{G}}{\rm{S}}+{\beta }_{2}{\rm{paternal}}\_{\rm{a}}{\rm{g}}{\rm{e}}\\ \,\,\,+\,{\beta }_{3}{\rm{c}}{\rm{h}}{\rm{i}}{\rm{l}}{\rm{d}}\_{\rm{m}}{\rm{e}}{\rm{a}}{\rm{n}}\_{\rm{R}}{\rm{D}}+{\beta }_{4}{\rm{f}}{\rm{a}}{\rm{t}}{\rm{h}}{\rm{e}}{\rm{r}}\_{\rm{m}}{\rm{e}}{\rm{a}}{\rm{n}}\_{\rm{R}}{\rm{D}}\\ \,\,\,+\,{\beta }_{5}{\rm{c}}{\rm{h}}{\rm{i}}{\rm{l}}{\rm{d}}\_{\rm{p}}{\rm{r}}{\rm{o}}{\rm{p}}\_{\rm{aligned}}+{\beta }_{6}{\rm{f}}{\rm{a}}{\rm{t}}{\rm{h}}{\rm{e}}{\rm{r}}\_{\rm{p}}{\rm{r}}{\rm{o}}{\rm{p}}\_{\rm{aligned}}\\ \,\,\,+\,{\beta }_{7}{\rm{c}}{\rm{h}}{\rm{i}}{\rm{l}}{\rm{d}}\_{\rm{s}}{\rm{n}}{\rm{v}}{\rm{s}}+{\beta }_{8}{\rm{f}}{\rm{a}}{\rm{t}}{\rm{h}}{\rm{e}}{\rm{r}}\_{\rm{s}}{\rm{n}}{\rm{v}}{\rm{s}}\\ \,\,\,+\,{\beta }_{9}{\rm{median}}\_{\rm{V}}{\rm{A}}{\rm{F}}+{\beta }_{10}{\rm{median}}\_{\rm{B}}{\rm{F}}\end{array}$$

Similarly, the maternal model was as follows:

$$\begin{array}{l}{\rm{dnSNVs}}\_{\rm{m}}{\rm{a}}{\rm{t}}{\rm{e}}{\rm{r}}{\rm{n}}{\rm{a}}{\rm{l}}={\beta }_{0}+{\beta }_{1}{\rm{m}}{\rm{a}}{\rm{t}}{\rm{e}}{\rm{r}}{\rm{n}}{\rm{a}}{\rm{l}}\_{\rm{P}}{\rm{G}}{\rm{S}}+{\beta }_{2}{\rm{m}}{\rm{a}}{\rm{t}}{\rm{e}}{\rm{r}}{\rm{n}}{\rm{a}}{\rm{l}}\_{\rm{a}}{\rm{g}}{\rm{e}}\\ \,\,\,+\,{\beta }_{3}{\rm{c}}{\rm{h}}{\rm{i}}{\rm{l}}{\rm{d}}\_{\rm{m}}{\rm{e}}{\rm{a}}{\rm{n}}\_{\rm{R}}{\rm{D}}+{\beta }_{4}{\rm{mother}}\_{\rm{m}}{\rm{e}}{\rm{a}}{\rm{n}}\_{\rm{R}}{\rm{D}}\\ \,\,\,+\,{\beta }_{5}{\rm{c}}{\rm{h}}{\rm{i}}{\rm{l}}{\rm{d}}\_{\rm{p}}{\rm{r}}{\rm{o}}{\rm{p}}\_{\rm{aligned}}+{\beta }_{6}{\rm{mother}}\_{\rm{p}}{\rm{r}}{\rm{o}}{\rm{p}}\_{\rm{aligned}}\\ \,\,\,+\,{\beta }_{7}{\rm{c}}{\rm{h}}{\rm{i}}{\rm{l}}{\rm{d}}\_{\rm{s}}{\rm{n}}{\rm{v}}{\rm{s}}+{\beta }_{8}{\rm{mother}}\_{\rm{s}}{\rm{n}}{\rm{v}}{\rm{s}}\\ \,\,\,+\,{\beta }_{9}{\rm{median}}\_{\rm{V}}{\rm{A}}{\rm{F}}+{\beta }_{10}{\rm{median}}\_{\rm{B}}{\rm{F}}\end{array}$$

Finally, as a check, we assessed the association between the maternal PGS and paternally phased dnSNVs, and vice versa, using a Poisson regression with an identity link where the same starting value for coefficients were supplied:

$$\begin{array}{l}{\rm{dnSNVs}}\_{\rm{p}}{\rm{a}}{\rm{t}}{\rm{e}}{\rm{r}}{\rm{n}}{\rm{a}}{\rm{l}}={\beta }_{0}+{\beta }_{1}{\rm{m}}{\rm{a}}{\rm{t}}{\rm{e}}{\rm{r}}{\rm{n}}{\rm{a}}{\rm{l}}\_{\rm{P}}{\rm{G}}{\rm{S}}+{\beta }_{2}{\rm{p}}{\rm{a}}{\rm{t}}{\rm{e}}{\rm{r}}{\rm{n}}{\rm{a}}{\rm{l}}\_{\rm{a}}{\rm{g}}{\rm{e}}\\ \,\,\,+\,{\beta }_{3}{\rm{c}}{\rm{h}}{\rm{i}}{\rm{l}}{\rm{d}}\_{\rm{m}}{\rm{e}}{\rm{a}}{\rm{n}}\_{\rm{R}}{\rm{D}}+{\beta }_{4}{\rm{f}}{\rm{a}}{\rm{t}}{\rm{h}}{\rm{e}}{\rm{r}}\_{\rm{m}}{\rm{e}}{\rm{a}}{\rm{n}}\_{\rm{R}}{\rm{D}}\\ \,\,\,+\,{\beta }_{5}{\rm{c}}{\rm{h}}{\rm{i}}{\rm{l}}{\rm{d}}\_{\rm{p}}{\rm{r}}{\rm{o}}{\rm{p}}\_{\rm{a}}{\rm{l}}{\rm{i}}{\rm{g}}{\rm{n}}{\rm{e}}{\rm{d}}+{\beta }_{6}{\rm{f}}{\rm{a}}{\rm{t}}{\rm{h}}{\rm{e}}{\rm{r}}\_{\rm{p}}{\rm{r}}{\rm{o}}{\rm{p}}\_{\rm{a}}{\rm{l}}{\rm{i}}{\rm{g}}{\rm{n}}{\rm{e}}{\rm{d}}\\ \,\,\,+\,{\beta }_{7}{\rm{c}}{\rm{h}}{\rm{i}}{\rm{l}}{\rm{d}}\_{\rm{s}}{\rm{n}}{\rm{v}}{\rm{s}}+{\beta }_{8}{\rm{f}}{\rm{a}}{\rm{t}}{\rm{h}}{\rm{e}}{\rm{r}}\_{\rm{s}}{\rm{n}}{\rm{v}}{\rm{s}}\\ \,\,\,+\,{\beta }_{9}{\rm{m}}{\rm{e}}{\rm{d}}{\rm{i}}{\rm{a}}{\rm{n}}\_{\rm{V}}{\rm{A}}{\rm{F}}+{\beta }_{10}{\rm{m}}{\rm{e}}{\rm{d}}{\rm{i}}{\rm{a}}{\rm{n}}\_{\rm{B}}{\rm{F}}\end{array}$$

$$\begin{array}{l}{\rm{dnSNVs}}\_{\rm{maternal}}={\beta }_{0}+{\beta }_{1}{\rm{paternal}}\_{\rm{P}}{\rm{G}}{\rm{S}}+{\beta }_{2}{\rm{maternal}}\_{\rm{a}}{\rm{g}}{\rm{e}}\\ \,\,\,+\,{\beta }_{3}{\rm{c}}{\rm{h}}{\rm{i}}{\rm{l}}{\rm{d}}\_{\rm{m}}{\rm{e}}{\rm{a}}{\rm{n}}\_{\rm{R}}{\rm{D}}+{\beta }_{4}{\rm{mother}}\_{\rm{m}}{\rm{e}}{\rm{a}}{\rm{n}}\_{\rm{R}}{\rm{D}}\\ \,\,\,+\,{\beta }_{5}{\rm{c}}{\rm{h}}{\rm{i}}{\rm{l}}{\rm{d}}\_{\rm{p}}{\rm{r}}{\rm{o}}{\rm{p}}\_{\rm{aligned}}+{\beta }_{6}{\rm{mother}}\_{\rm{p}}{\rm{r}}{\rm{o}}{\rm{p}}\_{\rm{aligned}}\\ \,\,\,+\,{\beta }_{7}{\rm{c}}{\rm{h}}{\rm{i}}{\rm{l}}{\rm{d}}\_{\rm{s}}{\rm{n}}{\rm{v}}{\rm{s}}+{\beta }_{8}{\rm{mother}}\_{\rm{s}}{\rm{n}}{\rm{v}}{\rm{s}}\\ \,\,\,+\,{\beta }_{9}{\rm{median}}\_{\rm{V}}{\rm{A}}{\rm{F}}+{\beta }_{10}{\rm{median}}\_{\rm{B}}{\rm{F}}\end{array}$$

### Rare variant burden in ANM genes in 100kGP

We tested for association between the burden of rare coding variants in ANM-associated genes in mothers and fathers with phased de novo SNVs in their offspring. We extracted high-confidence PTVs annotated by LOFTEE in the nine genes associated with ANM at the exome-wide significance (Fig. 2), as well as damaging missense variants with CADD > 25 in *CHEK2* and *SAMHD1*. Annotations were extracted from VEP105. All variants had MAF < 0.1% in the 100kGP aggV2 dataset and in all sub-populations in gnomAD. We set to missing genotypes with depth <7 and genotype quality <20 or heterozygous genotypes with a binomial test *P* < 0.001.

We first regressed out the same covariates as described above in the PGS analysis from maternally phased DNMs using a linear regression. We applied a rank-based inverse normal transformation on the residuals and fitted a linear regression model of the adjusted DNMs on carrier status in unrelated mothers for each of the 9 genes that were associated with ANM, adjusting for 20 genetic principal components. We adjusted paternally phased DNMs and regressed it on gene burden similarly in unrelated fathers.

### Mendelian randomization

#### Instrumental variable selection

Mendelian randomization analysis was applied to test whether the common variants associated with ANM^1^ have a causal effect on DNM rates in the offspring (Supplementary Table 15). In this approach, genetic variants that are significantly associated with an exposure, in this case ANM, are used as instrumental variables to test the causality of that exposure on the outcome of interest, in this case DNM rate^84–86^. For a genetic variant to be a reliable instrument, the following assumptions should be met: (1) the genetic instrument is associated with the exposure of interest; (2) the genetic instrument should not be associated with any other competing risk factor that is a confounder; and (3) the genetic instrument should not be associated with the outcome, except via the causal pathway that includes the exposure of interest^84,87^. Genotypes at all variants were aligned to designate the ANM PGS-increasing alleles as the effect alleles as described above and this was used as a genetic instrument of interest. The effect sizes of genetic instruments (genotypes in the mother) on maternally phased de novo SNVs in the offspring estimated in 8,089 duos were obtained from Genomics England.

#### Mendelian randomization frameworks

The Mendelian randomization analysis was conducted using the inverse-variance weighted (IVW) model as the primary model due to the highest statistical power^88^. However, as it does not correct for heterogeneity in outcome risk estimates between individual variants^89^, we applied a number of sensitivity Mendelian randomization methods that better account for heterogeneity^90^. These include Mendelian randomization Egger to identify and correct for unbalanced heterogeneity (‘horizontal pleiotropy’), indicated by a significant Egger intercept (*P* < 0.05)^91^, and weighted median (WM) and penalized weighted median (PWM) models to correct for balanced heterogeneity^92^. In addition, we introduced the Mendelian randomization radial method to exclude variants from each model in cases where they are recognized as outliers^93^. The results were considered as significant based on the *P* value significance consistency across different primary and sensitivity models applied. The results are available in Supplementary Table 15.

### Analyses of DNMs in deCODE

#### Identifying DNMs

The genome of the Icelandic population was characterised by whole-genome sequencing of 63,460 Icelanders using Illumina standard TruSeq methodology to a mean depth of 35 × (s.d. 8×) with subsequent long-range phasing^16^. Analyses of DNMs were restricted to individuals with at least 20× coverage.

DNM candidates were called in 9,643 trios in a similar manner to that described previously^32,33^, by comparing the genotypes of the parents and offspring. In brief, we defined a DNM candidate with permissive cut-offs for the genotype of the proband requiring that allele balance is greater than 25% and that there be at least 12 reads at the position (supporting either the reference or alternative allele). For the genotypes of the parents, we required at least 12 reads, maximum of one read supporting the alternative allele and the allelic balance to be less than 5%. Likely (*N*_LIK_) and possible carriers (*N*_POSS_) of the DNM allele outside the descendants of the parent pair were defined as before^32^. We restricted to DNM candidates with fewer than 50 likely carriers and either fewer than 10 possible carriers or with a ratio *N*_LIK_/*N*_POSS_ greater than 80%.

We tuned the DNM candidate filtering by using segregation of DNM candidates in three-generation families (2,042 probands), following^32^. We restricted to instances of the DNM candidates where we see both of the proband’s haplotypes at a locus transmitted to the offspring of the proband (see fig. 1c in ref. ^32^). In brief, if the DNM is a true germline variant then the allele of the DNM candidate should be present in the offspring who inherited the haplotype on which it lies. On the other hand, if it is absent from the children, despite both the haplotypes of the proband having been transmitted to at least one offspring, this suggests that the DNM candidate is a false-positive DNM call (see more detailed description in ref. ^32^). As before^32^, we fitted the generalized additive model with a logistic link using the mgcv R package^94^, using various functions of the following quality metrics as covariates, as indicated in the code:

- AAScore: prediction probability from Graphtyper that the variant is a true positive.
- Carrier_regression_beta: slope from the alternative allele depth regression for the sequence variant (described in the burden method section from deCODE).
- Carrier_regression_alpha: intercept from the alternative allele depth regression for the sequence variant (described in the burden method section from deCODE).
- Proband_het_AB: the allelic balance of the proband.
- MaxAAS: the maximum read support for the sequence variant across all individuals.
- Alignment_Alt_Reads: the number of reads supporting the alternative allele. This covariate and the following covariates were derived by identifying the reads in the BAM files supporting the de novo allele.
- Alignment_Alt_Unique_Positions: the unique number of starting positions for the reads supporting the alternative allele.
- Alignment_Alt_Soft_clipped: the number of soft clipped bases (S in CIGAR string).
- Alignment_Alt_Matched_bases: the number of matched bases (M in CIGAR string).
- Alignment_Alt_Score_diff: the difference in the alignment score between the best and the second best hit as reported by BWA mem.
- Alignment_Alt_Pair_sw_nm: the pairwise mismatches between reads supporting the alternative allele using the Smith Waterman implementation in SeqAn^95^.
- Alignment_Alt_Pair_align: the number of bases in the pairwise alignments.

We fitted the following formula within the gam() function in the mgcv R package^94^:


threegen_Consistent_hs~I(cut(alignment_Alt_Unique_Positions,c(−1,2,4,8,10,Inf)))+s(I(AAScore))+s(Carrier_regression_beta)+s(Carrier_regression_alpha)+I(ifelse(alignment_Alt_Reads>0,(alignment_Alt_Score_diff/alignment_Alt_Reads)>10,FALSE))+I(ifelse(alignment_Alt_Pair_align>0,(alignment_Alt_Pair_sw_nm/alignment_Alt_Pair_align)>0.05,TRUE))+I(ifelse(alignment_Alt_Matched_bases>0,alignment_Alt_Soft_clipped/alignment_Alt_Matched_bases>0.5,TRUE))+s(Proband_het_AB)+s(ifelse(MaxAAS>15,16,MaxAAS))+


I(NPOSS = = 0)

We then took the model learned using informative DNMs in these three-generation families and applied it to all remaining DNM candidates. We retained candidate DNMs for which the predicted probability of being a real DNM based on this model was at least 50%.

To validate the false-positive detection rate of the DNMs, we also used the genotype consistency between pairs of monozygotic twins. We found that 3.8% of DNMs are unobserved in the monozygotic twin of the proband. These could either be false-positive DNM calls or high frequency post-zygotic mutations that differ between pairs of monozygotic twins^96^.

To mirror the analysis of 100kGP, we phased the DNMs using read-backed phasing as previously described^32^.

#### Associating the ANM PGS with DNMs in deCODE

We calculated the raw ANM PGS per individual by multiplying the effect estimate by the count of the effect allele and summing the product across SNPs. We rank-transformed the raw PGS distribution across individuals to a standardized normal distribution. We fitted the following Poisson regression with identity link to assess the association between the mother’s PGS and the number of maternally phased DNMs:

$$\begin{array}{l}{\rm{dnSNVs}}\_{\rm{m}}{\rm{a}}{\rm{t}}{\rm{e}}{\rm{r}}{\rm{n}}{\rm{a}}{\rm{l}} \sim {\rm{maternal}}\_{\rm{P}}{\rm{G}}{\rm{S}}+{\rm{m}}{\rm{a}}{\rm{t}}{\rm{e}}{\rm{r}}{\rm{n}}{\rm{a}}{\rm{l}}\_{\rm{a}}{\rm{g}}{\rm{e}}\\ \,\,\,+\,{\rm{p}}{\rm{a}}{\rm{t}}{\rm{e}}{\rm{r}}{\rm{n}}{\rm{a}}{\rm{l}}\_{\rm{c}}{\rm{o}}{\rm{v}}{\rm{e}}{\rm{r}}{\rm{a}}{\rm{g}}{\rm{e}}\,+\,{\rm{maternal}}\_{\rm{c}}{\rm{o}}{\rm{v}}{\rm{e}}{\rm{r}}{\rm{a}}{\rm{g}}{\rm{e}}\\ \,\,\,+\,{\rm{c}}{\rm{h}}{\rm{i}}{\rm{l}}{\rm{d}}\_{\rm{c}}{\rm{o}}{\rm{v}}{\rm{e}}{\rm{r}}{\rm{a}}{\rm{g}}{\rm{e}}\,+\,{\rm{G}}{\rm{A}}{\rm{M}}\_{\rm{P}}{\rm{r}}{\rm{e}}{\rm{d}}{\rm{i}}{\rm{c}}{\rm{t}}\_{\rm{M}}{\rm{e}}{\rm{a}}{\rm{n}}\end{array}$$

where GAM_Predict_Mean is the average probability of the DNMs in the probands being real, calculated using the method described above.

We also fitted the following models as negative controls:

$$\begin{array}{l}{\rm{dnSNVs}}\_{\rm{paternal}} \sim {\rm{paternal}}\_{\rm{P}}{\rm{G}}{\rm{S}}+{\rm{paternal}}\_{\rm{a}}{\rm{g}}{\rm{e}}\\ \,\,\,+\,{\rm{paternal}}\_{\rm{coverage}}+{\rm{maternal}}\_{\rm{coverage}}\\ \,\,\,+{\rm{c}}{\rm{h}}{\rm{i}}{\rm{l}}{\rm{d}}\_{\rm{coverage}}+{\rm{G}}{\rm{A}}{\rm{M}}\_{\rm{Predict}}\_{\rm{M}}{\rm{e}}{\rm{a}}{\rm{n}}\end{array}$$

$$\begin{array}{l}{\rm{dnSNVs}}\_{\rm{paternal}} \sim {\rm{maternal}}\_{\rm{P}}{\rm{G}}{\rm{S}}+{\rm{paternal}}\_{\rm{a}}{\rm{g}}{\rm{e}}\\ \,\,\,+\,{\rm{paternal}}\_{\rm{coverage}}+{\rm{materna}}\_{\rm{coverage}}\\ \,\,\,+{\rm{c}}{\rm{h}}{\rm{i}}{\rm{l}}{\rm{d}}\_{\rm{coverage}}+{\rm{G}}{\rm{A}}{\rm{M}}\_{\rm{P}}{\rm{r}}{\rm{e}}{\rm{d}}{\rm{i}}{\rm{c}}{\rm{t}}\_{\rm{M}}{\rm{e}}{\rm{a}}{\rm{n}}\end{array}$$

To fit these models, we randomly chose one offspring per family.

#### ANM genetic variants and DNMs in deCODE

We analysed variants with MAF < 2%. This was a higher threshold than had been used in 100kGP since our analyses of ANM associations indicated that this threshold appeared to be better powered within deCODE, possibly because some deleterious variants have risen to a higher frequency due to the Icelandic bottleneck. We focused on PTVs in the nine genes associated with ANM at the exome-wide significance (Fig. 2), as well as damaging missense variants with CADD > 25 in *CHEK2* and *SAMHD1*.

For maternally and paternally phased DNMs, we adjusted the number of DNM for parental ages at conception and normalized the trait using rank-based inverse normal transformation. A linear regression model was used to test for association between the transformed DNM rate and the burden genotypes, assuming the variance-covariance matrix to be proportional to the kinship matrix.

We used an inverse-variance weighted approach to meta-analyse the results from 100kGP and deCODE. A Bonferroni correction of 18 tests (9 genes and 2 parents) was applied.

### Reporting summary

Further information on research design is available in the Nature Portfolio Reporting Summary linked to this article.

## Online content

Any methods, additional references, Nature Portfolio reporting summaries, source data, extended data, supplementary information, acknowledgements, peer review information; details of author contributions and competing interests; and statements of data and code availability are available at 10.1038/s41586-024-07931-x.

## Supplementary information

Supplementary Information This file contains a Supplementary Note, extended participant list, and extended acknowledgements.

Reporting Summary

Supplementary Tables Supplementary Tables 1–18

## Data Availability

All data used in discovery analyses are available upon application from the UK Biobank study and Genomics England. Research on the de-identified patient data used in this publication can be carried out in the Genomics England Research Environment subject to a collaborative agreement that adheres to patient led governance. All interested readers will be able to access the data in the same manner that the authors accessed the data. For more information about accessing the data, interested readers may contact research-network@genomicsengland.co.uk or access the relevant information on the Genomics England website: https://www.genomicsengland.co.uk/research. The deCODE dataset was used for replication purposes and only summary level results for the specific findings are provided.
