# Supplementary material for: Genetic links between ovarian ageing, cancer risk and de novo mutation rates
Source: Nature. 2024 Sep 11;633(8030):608–14. doi: 10.1038/s41586-024-07931-x (PMC11410666; doi:10.1038/s41586-024-07931-x)
Supplement: Supplementary file 1 — This file contains a Supplementary Note, extended participant list, and extended acknowledgements. [file 41586_2024_7931_MOESM1_ESM.docx]

Supplementary Material for

**Genetic links between ovarian ageing, cancer risk and *de novo* mutation rates**

Stasa Stankovic*^1^, Saleh Shekari*^2,3^, Qin Qin Huang*^4^, Eugene J. Gardner*^1^, Erna V. Ivarsdottir*^5^, Nick D. L. Owens*^2^, Nasim Mavaddat^6^, Ajuna Azad^7^,Gareth Hawkes^2^, Katherine A. Kentistou^1^, Robin N. Beaumont^2^, Felix R. Day^1^, Yajie Zhao^1^, Hakon Jonsson^5^, Thorunn Rafnar^5^, Vinicius Tragante^5^, Gardar Sveinbjornsson^5^, Asmundur Oddsson^5^, Unnur Styrkarsdottir^5^, Julius Gudmundsson^5^, Simon N. Stacey^5^, Daniel F. Gudbjartsson^5^, Breast Cancer Association Consortium, Kitale Kennedy^2^, Andrew R. Wood^2^, Michael N. Weedon^2^, Ken K. Ong^1,8^, Caroline F. Wright^2^, Eva R. Hoffmann^7^, Patrick Sulem^5^, Matthew E. Hurles^4^, Katherine S. Ruth^2^, Hilary C. Martin*^4^, Kari Stefansson*^5^, John R. B. Perry*^1,9^ and Anna Murray*^2^

* Denotes equal contribution

Correspondence to:

John R.B Perry ([john.perry@mrc-epid.cam.ac.uk](mailto:john.perry@mrc-epid.cam.ac.uk))

Anna Murray ([A.Murray@exeter.ac.uk](mailto:A.Murray@exeter.ac.uk))

Table of Contents

[Supplementary Note 2](#_Toc171931706)

[Comparison with previously published results on ANM WES 2](#_Toc171931707)

[Associations not captured in current analysis 2](#_Toc171931708)

[Associations not captured by Ward et al. 2](#_Toc171931709)

[Common ANM associated variants are enriched in *ZNF518A* binding sites 3](#_Toc171931710)

[Functional enrichment tests for *ZNF518A* transcription factor binding sites using fGWAS and SLDP 5](#_Toc171931711)

[Functional analysis of ZNF518A binding sites 6](#_Toc171931712)

[Extended authorship list 7](#_Toc171931713)

[Extended acknowledgements 10](#_Toc171931714)

# **Supplementary Note**

## **Comparison with previously published results on ANM WES**

A previous ANM analysis of the 450K UKBB exome data, published by Ward and colleagues, identified seven genes: *CHEK2*, *DCLRE1A*, *HELB*, *CLPB*, *TOP3A*, *RAD54L* and *HROB*^5^. Our results replicate the association with *CHEK2* and *HELB* and provide more robust evidence (*P* = 1.9*10^-8^) for the previously described suggestive *HROB* association (*P* = 2.9*10^-6^). In addition, we identified six genes, which were not captured by Ward *et al*.: *ZNF518A*, *BRCA2*, *ETAA1*, *PALB2*, *PNPLA8* and *SAMHD1* (**Supplementary Table 5**). We investigated potential study design differences that could account for variation in the findings as the same data was used in both studies.

## **Associations not captured in current analysis**

First, we investigated potential analytical parameters that could account for differences in the findings. Associations with *DCLRE1A*, *RAD54L*, *TOP3A* and *CLPB* were not identified in our study, because we restricted our analysis to variants with a MAF <0.1%, rather than <1%. We re-analysed our data with a burden test MAF threshold of <1% and three of the four associations were replicated: *DCLRE1A* (*P* _MAF 1%_= 3.8*10^-8^, N=1056), *RAD54L* (*P* _MAF 1%_= 6.4*10^-7^, N=1892) and *TOP3A* (*P* _MAF 1%_= 1.5*10^-7^, N=2001). *RAD54L and* *TOP3A* were genes highlighted by GWAS^1^ and the exome association in *TOP3A* was driven by a single, relatively common variant (rs34001746, MAF=0.7%, *P*=1.63*10^-10^). This variant was in LD with the previously reported lead GWAS SNP (rs569145577, r^2^=0.92), with little evidence for association after its exclusion (*P*=0.50 for all other missense and PTVs). *CLPB* just missed our *P*-value threshold, but again a single variant (rs150343959, *P*=8.22*10^-6^) was largely driving the association signal - when excluded in leave-one-out analysis, the *CLPB* burden association dropped (*P*=1.19*10^-2^). By including relatively common variants in gene burden masks, single variants can dominate the general functional effect being tested, which could be contributed to by LD with non-exomic functional variants. Therefore, in order to be able to make a stronger link between genetic variants and individual genes, we chose to restrict our analysis to rarer variants with MAF <0.1%.

## **Associations not captured by Ward et al.**

Differences in MAF thresholds did not explain why our study identified an additional six genes (*BRCA2*, *ETAA1*, *PALB2*, *PNPLA8*, *SAMHD1* and *ZNF518A*) compared with Ward *et al.*^5^. We therefore tested differences in the phenotype preparation, tools and variant masks used to test the associations. Four of our six gene burden associations (*BRCA2*, *PALB2*, *PNPLA8*, and *SAMHD1*) were relatively near the borderline of the significance threshold in our analyses in the primary BOLT-LMM pipeline, although *ETAA1* and *PALB2* were just below the threshold in the REGENIE pipeline (**Supplementary Table 5**).

We had a ~20% larger sample size (106,973 post-menopausal women) in comparison to Ward *et al*.^5^ (78,311 unrelated post-menopausal women), which would have resulted in more statistical power in our analyses (**Supplementary Table 5**). This was particularly important for *BRCA2*, *ETAA1* and *PALB2* - Ward *et al.* included 63 (19.5%) fewer *BRCA2* and 46 (21.7%) fewer *PALB2* carriers of rare damaging variants (Ward *et al.* REGENIE analysis vs. our main analysis) and identified the ANM association at these genes only at the borderline of exome-wide significance, *P*=1.55*10^-6^ and *P*=7.47*10^-5^, respectively. Similarly, for *SAMHD1*, Ward *et al.* captured 57 (24.3%) fewer carriers in their linear regression model compared with our main analysis, which resulted in association *P* values of 6.38*10^-4^ in the linear regression model and *P*=8.02*10^-6^ in the time to event analysis. Ward *et al.* used only unrelated individuals in their primary analyses, where we used linear mixed models and were therefore able to include an additional ~19,000 related individuals. Secondly, Ward *et al.* excluded ~2,300 women with ANM <40 and >60 years, while we used the full natural menopause distribution. Finally, the difference in sample size was partly due to differences in phenotype preparation (resulting in an additional ~7,400 women); specifically, we considered four instances where questions regarding ANM were asked, whereas Ward *et al.* used data from the baseline visit in their main analysis.

As sensitivity analyses and to better replicate the methods of Ward *et al.*, for four of the genes (*CHEK2* and *DCLRE1A* found in both analyses, and the novel associations in *ZNF518A* and *PNPLA8*) we compared results from linear regression in unrelated individuals using MAF<1% with those from a truncated menopause distribution (ANM 40-60 years) and a time-to-event Cox proportional hazards model (**Supplementary Table 5**). For the truncated distribution, all four genes passed the threshold of exome-wide association and, for all with the exception of *ZNF518A*, the association *P* value was larger (*CHEK2*: 3.3*10^-35^, *DCLRE1A*: 1.3*10^-7^, *ZNF518A*: 8.4*10^-11^, *PNPLA8*: 3*10^-8^) than for analyses based on the full range of ANM. Association statistics from the Cox model (*CHEK2*: 2.4*10^-39^, *DCLRE1A*: 6*10^-8^, *ZNF518A*: 1.4*10^-9^, *PNPLA8*: 5.7*10^-10^) were comparable to those from linear regression models based on the full range of ANM (*CHEK2*: 3.1*10^-46^, *DCLRE1A*: 2.5*10^-8^, *ZNF518A*: 1.2*10^-9^, *PNPLA8*: 1.9*10^-9^).

Finally, differences in variant annotation may also explain some inconsistencies between studies. *ZNF518A* was not reported by Ward *et al.*, which may be because all variants are in the last and only coding exon of the gene, and in some annotations such variants would inappropriately be excluded from being considered as loss of function. We note that another single coding exon gene (*NFIL3*) was not included in the Ward *et al.* publication but was in our analysis. Detailed comparisons between our study and Ward *et al.* are available in **Supplementary Table 5**.

## **Common ANM associated variants are enriched in *ZNF518A* binding sites**

Heterozygous loss-of-function of *ZNF518A* had the largest effect on ANM of the genes we identified. *ZNF518A* is a poorly characterised C2H2 zinc finger transcription factor, which has been shown to associate with PRC2 and G9A-GLP repressive complexes along with its paralog *ZNF518B*, suggesting a potential role in transcriptional repression^19^. *ZNF518A* localises robustly to 18,706 sites in the genome, based on ChIP-seq data available from ENCODE^20,21^ and binds primarily to gene promoters, with 33.5% (6,263) of *ZNF518A* binding sites within 2kb of a transcription start site (TSS) (**Extended Data Figure 3a-c**). Common variants associated with ANM^1^ were enriched in the transcriptional targets of *ZNF518A* (*P*=1.32*10^-4^) using fGWAS^24^. We further tested functional enrichment using signed linkage disequilibrium profile (SLDP) regression^25^. This confirmed the enrichment of *ZNF518A* binding sites near to loci associated with ANM and showed that its transcriptional repression is associated with earlier ANM (*P*=0.02), consistent with evidence from rare variant burden tests. Separating *ZNF518A* sites by those proximal (< 2Kb) and distal (>5kb) from a TSS, demonstrated this association was due to *ZNF518A* binding at regulatory regions distal to the TSS (proximal TSS *P*=0.3, distal *ZNF518A* *P*=0.002). Notably, these regulatory *ZNF518A* bound loci produce the largest association amongst an SLDP catalogue of 382 transcription factors and regulators (**Supplementary Table 7, Extended Data Figure 3d**). These results suggest a different functional role for *ZNF518A* at TSS and more distal regulatory regions. In order to explore this further we assessed the sequence determinants of *ZNF518A* binding. *De novo* motif discovery identified an AT-rich motif enriched at distal regulatory *ZNF518A* binding sites, but not at TSS bound by *ZNF518A*. This AT-rich motif was centrally enriched within *ZNF518A* ChIP-seq peaks and matched an unvalidated motif present in the JASPAR transcription factor motif database^97^ (**Extended Data Figure 3e**). We found the number of perfect instances of this AT-rich motif to be strongly associated with *ZNF518A* occupancy as assessed by *ZNF518A* ChIP-seq signal at distal regions but not at TSS (**Extended Data Figure 3f,g**). At distal regions, the maximal association between peaks greater than the median height was found at least seven motif instances (hypergeometric right tail *P* < 10^-389^, OR 7.41). These data suggest that *ZNF518A* is recruited by DNA sequence at distal sites, but at TSS may be recruited to gene promoters by interaction with another DNA binding factor.

We next employed public *in vitro* differentiated human primordial germ like-cell data^98,99^ to assess the chromatin state at *ZNF518A* bound loci, directly comparing distal regions with TSS. *ZNF518A* bound TSS showed chromatin accessibility^99^ and were marked with H3K27ac^98^. In contrast, distal regions lacked H3K27ac and showed minimal chromatin accessibility **(Extended Data Figure 3h)**. Extending this comparison to the Epimap chromatin states^100^, we find that overall *ZNF518A* bound loci are enriched in active TSS and that distal *ZNF518A* regions are variously enriched in active and repressed chromatin **(Extended Data Figure 3i,j)**. Consistent with previous data which has found *ZNF518A* in repressive complexes, these data suggest that *ZNF518A* is recruited by DNA sequence to distal regulatory regions where it acts to repress local chromatin.

While *ZNF518A* is known to have diverse tissue expression including the ovary, we found that it was highly expressed in fetal germ cells at both the mitotic and meiotic stages (**Extended Data Figures 6 and 7, Supplementary Tables 17 & 18**). The eight other WES genes identified in this study were expressed at varying levels in fetal gonadal cells, oocytes and granulosa cells across different developmental stages (**Extended Data Figures 6 and 7, Supplementary Tables 17 & 18**).

## **Functional enrichment tests for *ZNF518A* transcription factor binding sites using fGWAS and SLDP**

fGWAS (*v.0.3.6)*, a hierarchical model for joint analysis of GWAS and genomic annotations, was implemented to test the functional enrichment of ANM GWAS hits in *ZNF518A* transcription factor binding sites^24^. The fGWAS input file contained the ANM GWAS summary stats derived from the Reprogen study^1^ annotated for *ZNF518A* binding sites. The *ZNF518A* annotation file was derived from the ENCODE ChIP-seq data^20,21^ from human HEK293 cell line^101^ the optimal independent discovery rate peak calling against hg19 [ENCFF415VBF] was used. The ANM GWAS hits were annotated for the presence/absence of the *ZNF518A* transcription factor binding sites in a binary way (0, 1), with ‘1’ if the SNP falls within the transcription factor binding site and ‘0’ otherwise. The fGWAS tool, available from <https://github.com/joepickrell/fgwas>, was run in annotation mode “-w” for the described *ZNF518A* annotation. Detailed description of fGWAS methodology is available in Pickrell *et al*, 2014^24^. In short, the genome is split into independent blocks, which are allowed to contain either a single polymorphism that causally influences the trait or none. fGWAS then models the prior probability that any given block contains an association and the conditional prior probability that any given SNP in the block is the causal one, with probabilities allowed to vary according to functional annotations. The priors are then estimated using an empirical Bayes approach. The fGWAS output contained the maximum likelihood parameter estimates for each parameter in the model, in this case *ZNF518A*, with the lower and upper bound of the 95% CI on the parameter. The *P* value was calculated from lower and upper CI following methodology described in Altman *et al*, 2011^102^, in 3 following steps: (1) Standard error (SE) calculation: *SE* = (*Upper CI* − *Lower CI*)/(2*1.96); (2) Test statistics calculation: *Z=Estimate / SE*; and (3) P value calculation: *P = exp(−0.717*Z − 0.416*Z^2^)*.

Signed LD profile (SLDP) regression was applied to explore the directional effect of a signed functional annotation, *ZNF518A*, on a heritable trait like ANM using GWAS summary statistics^1^. More specifically, we tested whether alleles that are predicted to increase the binding of the transcription factor *ZNF518A* have a genome-wide tendency to increase or decrease timing of menopause in women. The SLDP tool was installed from <https://github.com/yakirr/sldp>, with the comprehensive methodological steps described in Reshef *et al*, 2018^25^. For the analysis to be conducted, SLDP required GWAS summary statistics for ANM, signed LD profiles for *ZNF518A* binding, signed background model and reference panel in a SLDP compatible format. For the reference we used a 1000 Genomes Phase 3 European reference panel in *plink* format, which contained approximately 10M SNPs and 500 people and was available for download at the ‘[refpanel](https://alkesgroup.broadinstitute.org/SLDP/refpanel/)’ page. The ANM GWAS summary statistics, available from our latest Reprogen study^1^, was pre-processed using the ‘*preprocesspheno*’ tool from the SLDP package. To conduct this step, we also obtained the list of regression SNPs along with the LD scores for the reference panel from the ‘[refpanel](https://alkesgroup.broadinstitute.org/SLDP/refpanel/)’ page. The pre-processing step included filtering down to SNPs that are also present in the reference panel, harmonising alleles to the reference, and multiplying the summary statistics by the SLDP regression weights. In addition, we applied the ‘*preprocessrefpanel*’ tool to compute a truncated singular value decomposition (SVD) for each LD block in the reference panel. These SVDs were later used to weight the SLDP regression. The *ZNF518A* annotation file was obtained from the ENCODE CHIP-seq analysis^20,21^, as described above, and preprocessed using the ‘*preprocessannot*’ tool that turns signed functional annotations into signed LD profiles. Prior to running SLDP, we also obtained the signed background LD profiles that enabled us to control for systematic signed effects of minor alleles, which could arise from either population stratification or negative selection. SLDP was then run on our data using ‘*sldp*’ function. To explore the relevance of *ZNF518A* for menopause timing in comparison to other transcription regulators, we tested whether genome-wide sequence changes introduced by SNP alleles identified in ANM GWAS increase or decrease binding of additional 382 transcription factors (TFs). The preprocessed annotation files for 382 TFs derived from ENCODE CHIP-seq experiments^20,21^, were available for download at the [annotation data page](https://alkesgroup.broadinstitute.org/SLDP/annots/basset/). The results are available in **Supplementary Table 7**.

## **Functional analysis of ZNF518A binding sites**

*ZNF518A* peaks were derived from unique genomic regions in ENCODE accession ENCFF415VBF described above. Quantification of ChIP-seq signal by aligning paired-end replicates (ENCFF174HBR, ENCFF574GQY, ENCFF808AJP, ENCFF453FDD) to the hg19 genome with Bowtie2 v2.3.5.1^103^ with options “-I 0 -X 1000 –no-discordant –no-mixed”, reads were filtered for those with MAPQ > 30 with samtools v1.10. Assessment of H3K27ac^98^ and chromatin accessibility by ATAC-seq^99^ in day 4 human primordial germ cell like cells (hPGCLCs) at *ZNF518A* peaks was performed. For H3K27ac single end reads from accessions GSM4257216, GSM4257217, GSM4257218 were obtained and aligned with Bowtie2 v2.3.5.1 with default settings and MAPQ > 30 reads retained as above. For ATAC-seq paired-end reads were obtained from accessions GSM3406938, GSM3406939 and mapped and filtered as *ZNF518A* reads above.

Quantification of ChIP-seq and ATAC-seq signals for peak heights, heatmaps was performed with <https://github.com/owensnick/GenomeFragments.jl>. Peak to TSS distances were calculated against Gencode v36 release liftover to hg19 using GenomicFeatures.jl and <https://github.com/owensnick/ProximityEnrichment.jl>. We consider four categories of peaks: TSS intersecting, TSS proximal (TSS < 2000kb, outside gene body), Gene body intersecting, Intergenic and Distal (TSS > 5kb).

To perform *de novo* motif discovery we used Homer v4.11.1^104^ using findMotifsGenome.pl with options “hg19 -size 200”. We ran this on all *ZNF518A* peaks, distal peaks and those intersecting TSS, we recovered a motif matching JASPAR^97^ unvalidated motif UN0199.1 in all peak sets apart from those intersecting TSS. We then used <https://github.com/exeter-tfs/MotifScanner.jl> to quantify the occurrence of all instances of motif UN0199.1 in *ZNF518A* peaks.

We downloaded the 18-state ChromHMM^105^ models for all 833 biosamples in Epimap^100^ from <http://compbio.mit.edu/epimap/>. We calculated the intersection between each state in each biosample and either all *ZNF518A* peaks or distal *ZNF518A* peaks using GenomicFeatures.jl. We calculated odds ratios from contingency tables using the approximation of bedtools^106^ and Giggle^107^, by estimating total genomic intervals as hg19 genome size divided by the sum of the mean *ZNF518A* peak size and the chromatin state interval size.

# **Extended authorship list**

**Breast Cancer Association Consortium**

Mavaddat, N.^1^; Allen, J.^1^; Andrulis, I. L.^2, 3^; Bojesen, S. E.^4-6^; Bolla, M. K.^1^; Brauch, H.^7-9^; Carvalho, S.^1^; Castelao, J. E.^10^; Chang-Claude, J.^11, 12^; Czene, K.^13^; Devilee, P.^14, 15^; Dörk, T.^16^; Dorling, L.^1^; Dunning, A. M.^17^; Evans, D. G.^18, 19^; Fasching, P. A.^20^; Gago-Dominguez, M.^21^; García-Closas, M.^22^; González-Neira, A.^23^; Guénel, P.^24^; Hall, P.^13, 25^; Hamann, U.^26^; Hartman, M.^27-29^; Kvist, A.^30^; Lindblom, A.^31, 32^; Mannermaa, A.^33-35^; Milne, R. L.^36-38^; Muir, K.^39^; Panagiotidis, M. I.^40^; Pharoah, P. D. P.^1, 17^; Rashid, M. U.^26, 41^; Saloustros, E.^42^; Schmidt, M.^43-45^; Hwang Teo, S.^46, 47^; Torres, D.^26, 48^; Wang, Q.^1^; Easton, D. F.^1, 17^.

^1^ Centre for Cancer Genetic Epidemiology, Department of Public Health and Primary Care, University of Cambridge, Cambridge, UK.

^2^ Fred A. Litwin Center for Cancer Genetics, Lunenfeld-Tanenbaum Research Institute of Mount Sinai Hospital, Toronto, Ontario, Canada.

^3^ Department of Molecular Genetics, University of Toronto, Toronto, Ontario, Canada.

^4^ Copenhagen General Population Study, Herlev and Gentofte Hospital, Copenhagen University Hospital, Herlev, Denmark.

^5^ Department of Clinical Biochemistry, Herlev and Gentofte Hospital, Copenhagen University Hospital, Herlev, Denmark.

^6^ Faculty of Health and Medical Sciences, University of Copenhagen, Copenhagen, Denmark.

^7^ Dr. Margarete Fischer-Bosch-Institute of Clinical Pharmacology, Stuttgart, Germany.

^8^ iFIT-Cluster of Excellence, University of Tübingen, Tübingen, Germany.

^9^ German Cancer Consortium (DKTK) and German Cancer Research Center (DKFZ), Partner Site Tübingen, Tübingen, Germany.

^10^ Oncology and Genetics Unit, Instituto de Investigación Sanitaria Galicia Sur (IISGS), Xerencia de Xestion Integrada de Vigo-SERGAS, Vigo, Spain.

^11^ Division of Cancer Epidemiology, German Cancer Research Center (DKFZ), Heidelberg, Germany.

^12^ Cancer Epidemiology Group, University Cancer Center Hamburg (UCCH), University Medical Center Hamburg-Eppendorf, Hamburg, Germany.

^13^ Department of Medical Epidemiology and Biostatistics, Karolinska Institutet, Stockholm, Sweden.

^14^ Department of Pathology, Leiden University Medical Center, Leiden, the Netherlands.

^15^ Department of Human Genetics, Leiden University Medical Center, Leiden, the Netherlands.

^16^ Gynaecology Research Unit, Hannover Medical School, Hannover, Germany.

^17^ Centre for Cancer Genetic Epidemiology, Department of Oncology, University of Cambridge, Cambridge, UK.

^18^ Division of Evolution and Genomic Sciences, School of Biological Sciences, Faculty of Biology, Medicine and Health, University of Manchester, Manchester Academic Health Science Centre, Manchester, UK.

^19^ North West Genomics Laboratory Hub, Manchester Centre for Genomic Medicine, St Mary’s Hospital, Manchester University NHS Foundation Trust, Manchester Academic Health Science Centre, Manchester, UK.

^20^ Department of Gynecology and Obstetrics, Comprehensive Cancer Center Erlangen-EMN, Friedrich-Alexander University Erlangen-Nuremberg, University Hospital Erlangen, Erlangen, Germany.

^21^ Genomic Medicine Group, International Cancer Genetics and Epidemiology Group, Fundación Pública Galega de Medicina Xenómica, Instituto de Investigación Sanitaria de Santiago de Compostela (IDIS), Complejo Hospitalario Universitario de Santiago, SERGAS, Santiago de Compostela, Spain.

^22^ Division of Cancer Epidemiology and Genetics, National Cancer Institute, National Institutes of Health, Department of Health and Human Services, Bethesda, MD, USA.

^23^ Human Genotyping Unit-CeGen, Spanish National Cancer Research Centre (CNIO), Madrid, Spain.

^24^ Team 'Exposome and Heredity', CESP, Gustave Roussy, INSERM, University Paris-Saclay, UVSQ, Villejuif, France.

^25^ Department of Oncology, Södersjukhuset, Stockholm, Sweden.

^26^ Molecular Genetics of Breast Cancer, German Cancer Research Center (DKFZ), Heidelberg, Germany.

^27^ Saw Swee Hock School of Public Health, National University of Singapore and National University Health System, Singapore, Singapore.

^28^ Department of Surgery, National University Health System, Singapore, Singapore.

^29^ Department of Pathology, Yong Loo Lin School of Medicine, National University of Singapore, Singapore, Singapore.

^30^ Division of Oncology and Pathology, Department of Clinical Sciences Lund, Lund University, Lund, Sweden.

^31^ Department of Molecular Medicine and Surgery, Karolinska Institutet, Stockholm, Sweden.

^32^ Department of Clinical Genetics, Karolinska University Hospital, Stockholm, Sweden.

^33^ Translational Cancer Research Area, University of Eastern Finland, Kuopio, Finland.

^34^ Institute of Clinical Medicine, Pathology and Forensic Medicine, University of Eastern Finland, Kuopio, Finland.

^35^ Biobank of Eastern Finland, Kuopio University Hospital, Kuopio, Finland.

^36^ Cancer Epidemiology Division, Cancer Council Victoria, Melbourne, Victoria, Australia.

^37^ Centre for Epidemiology and Biostatistics, Melbourne School of Population and Global Health, The University of Melbourne, Melbourne, Victoria, Australia.

^38^ Precision Medicine, School of Clinical Sciences at Monash Health, Monash University, Clayton, Victoria, Australia.

^39^ Division of Population Health, Health Services Research and Primary Care, School of Health Sciences, Faculty of Biology, Medicine and Health, The University of Manchester, Manchester, UK.

^40^ Department of Cancer Genetics, Therapeutics and Ultrastructural Pathology, The Cyprus Institute of Neurology & Genetics, Nicosia, Cyprus.

^41^ Department of Basic Sciences, Shaukat Khanum Memorial Cancer Hospital and Research Centre (SKMCH & RC), Lahore, Pakistan.

^42^ Department of Oncology, University Hospital of Larissa, Larissa, Greece.

^43^ Division of Molecular Pathology, The Netherlands Cancer Institute, Amsterdam, the Netherlands.

^44^ Division of Psychosocial Research and Epidemiology, The Netherlands Cancer Institute - Antoni van Leeuwenhoek hospital, Amsterdam, the Netherlands.

^45^ Department of Clinical Genetics, Leiden University Medical Center, Leiden, the Netherlands.

^46^ Breast Cancer Research Programme, Cancer Research Malaysia, Subang Jaya, Selangor, Malaysia.

^47^ Department of Surgery, Faculty of Medicine, University of Malaya, UM Cancer Research Institute, Kuala Lumpur, Malaysia.

^48^ Institute of Human Genetics, Pontificia Universidad Javeriana, Bogota, Colombia.

#

#

# **Extended acknowledgements**

Stasa Stankovic was supported by the Clare Hall Ivan D Jankovic scholarship (University of Cambridge). Saleh Shekari was supported by the QUEX Institute (University of Exeter, UK and the University of Queensland, Australia). Anna Murray, Caroline Wright and Michael Weedon are supported by the Medical Research Council (MR/T00200X/1).The authors acknowledge the use of the University of Exeter High-Performance Computing facility in carrying out this work, funded by a MRC Clinical Research Infrastructure award (MRC Grant: MR/M008924/1). This study was supported by the National Institute for Health and Care Research Exeter Biomedical Research Centre. The views expressed are those of the author(s) and not necessarily those of the NIHR or the Department of Health and Social Care.

Ajuna Azad and Eva Hoffmann were supported by the ERC (724718-ReCAP), Novo Nordisk Foundation (NNF15COC0016662), the Independent Research Foundation Denmark (0134-00299B), and a grant from the Danish National Research Foundation Centre (6110-00344B).

This research was made possible through access to the data and findings generated by the 100,000 Genomes Project. The 100,000 Genomes Project is managed by Genomics England Limited (a wholly owned company of the Department of Health and Social Care). The 100,000 Genomes Project is funded by the National Institute for Health Research and NHS England. The Wellcome Trust, Cancer Research UK and the Medical Research Council have also funded research infrastructure. The 100,000 Genomes Project uses data provided by patients and collected by the National Health Service as part of their care and support.

BCAC is funded by the European Union's Horizon 2020 Research and Innovation Programme (grant numbers 634935 and 633784 for BRIDGES and B-CAST respectively), and the PERSPECTIVE I&I project, funded by the Government of Canada through Genome Canada and the Canadian Institutes of Health Research, the Ministère de l’Économie et de l'Innovation du Québec through Genome Québec, the Quebec Breast Cancer Foundation. The EU Horizon 2020 Research and Innovation Programme funding source had no role in study design, data collection, data analysis, data interpretation or writing of the report. Additional funding for BCAC is provided via the Confluence project which is funded with intramural funds from the National Cancer Institute Intramural Research Program, National Institutes of Health.

The BRIDGES panel sequencing was supported by the European Union Horizon 2020 research and innovation program BRIDGES (grant number, 634935) and the Wellcome Trust (v203477/Z/16/Z).

The **ABCS** study was supported by the Dutch Cancer Society [grants NKI 2007-3839; 2009 4363] and an institutional grant of the Dutch Cancer Society and of the Dutch Ministry of Health, Welfare and Sport. The **ACP** study is funded by the Breast Cancer Research Trust, UK. KM and AL are supported by the NIHR Manchester Biomedical Research Centre, the Allan Turing Institute under the EPSRC grant EP/N510129/1. The work of the **BBCC** was partly funded by ELAN-Fond of the University Hospital of Erlangen. The BREast Oncology GAlician Network (**BREOGAN**) is funded by Acción Estratégica de Salud del Instituto de Salud Carlos III FIS PI12/02125/Cofinanciado and FEDER PI17/00918/Cofinanciado FEDER; Acción Estratégica de Salud del Instituto de Salud Carlos III FIS Intrasalud (PI13/01136); Programa Grupos Emergentes, Cancer Genetics Unit, Instituto de Investigacion Biomedica Galicia Sur. Xerencia de Xestion Integrada de Vigo-SERGAS, Instituto de Salud Carlos III, Spain; Grant 10CSA012E, Consellería de Industria Programa Sectorial de Investigación Aplicada, PEME I + D e I + D Suma del Plan Gallego de Investigación, Desarrollo e Innovación Tecnológica de la Consellería de Industria de la Xunta de Galicia, Spain; Grant EC11-192. Fomento de la Investigación Clínica Independiente, Ministerio de Sanidad, Servicios Sociales e Igualdad, Spain; and Grant FEDER-Innterconecta. Ministerio de Economia y Competitividad, Xunta de Galicia, Spain. **CCGP** is supported by funding from the University of Crete. The **CECILE** study was supported by Fondation de France, Institut National du Cancer (INCa), Ligue Nationale contre le Cancer, Agence Nationale de Sécurité Sanitaire, de l'Alimentation, de l'Environnement et du Travail (ANSES), Agence Nationale de la Recherche (ANR). The **CGPS** was supported by the Chief Physician Johan Boserup and Lise Boserup Fund, the Danish Medical Research Council, and Herlev and Gentofte Hospital. **COLBCCC** is supported by the German Cancer Research Center (DKFZ), Heidelberg, Germany. Diana Torres was in part supported by a postdoctoral fellowship from the Alexander von Humboldt Foundation. **PROCAS** is funded from NIHR grant PGfAR 0707-10031. DGE, AH and WGN are supported by the NIHR Manchester Biomedical Research Centre (IS-BRC-1215-20007). The **GENICA** was funded by the Federal Ministry of Education and Research (BMBF) Germany grants 01KW9975/5, 01KW9976/8, 01KW9977/0 and 01KW0114, the Robert Bosch Foundation, Stuttgart, Deutsches Krebsforschungszentrum (DKFZ), Heidelberg, the Institute for Prevention and Occupational Medicine of the German Social Accident Insurance, Institute of the Ruhr University Bochum (IPA), Bochum, as well as the Department of Internal Medicine, Johanniter GmbH Bonn, Johanniter Krankenhaus, Bonn, Germany. The **GESBC** was supported by the Deutsche Krebshilfe e. V. [70492] and the German Cancer Research Center (DKFZ). The **HUBCS** was supported by a grant from the German Federal Ministry of Research and Education (RUS08/017), B.M. was supported by grant 17-44-020498, 17-29-06014 of the Russian Foundation for Basic Research, D.P. was supported by grant 18-29-09129 of the Russian Foundation for Basic Research, E.K was supported by the mega grant from the Government of Russian Federation (2020-220-08-2197), and the study was performed as part of the assignment of the Ministry of Science and Higher Education of the Russian Federation (№АААА-А16-116020350032-1). Financial support for **KARBAC** was provided through the regional agreement on medical training and clinical research (ALF) between Stockholm County Council and Karolinska Institutet, the Swedish Cancer Society, The Gustav V Jubilee foundation and Bert von Kantzows foundation. The **KARMA** study was supported by Märit and Hans Rausings Initiative Against Breast Cancer. The **KBCP** was financially supported by the special Government Funding (VTR) of Kuopio University Hospital grants, Cancer Fund of North Savo, the Finnish Cancer Organizations, and by the strategic funding of the University of Eastern Finland. The **MARIE** study was supported by the Deutsche Krebshilfe e.V. [70-2892-BR I, 106332, 108253, 108419, 110826, 110828], the Hamburg Cancer Society, the German Cancer Research Center (DKFZ) and the Federal Ministry of Education and Research (BMBF) Germany [01KH0402]. The **MASTOS** study was supported by “Cyprus Research Promotion Foundation” grants 0104/13 and 0104/17, and the Cyprus Institute of Neurology and Genetics. The Melbourne Collaborative Cohort Study (**MCCS**) cohort recruitment was funded by VicHealth and Cancer Council Victoria. The MCCS was further augmented by Australian National Health and Medical Research Council grants 209057, 396414 and 1074383 and by infrastructure provided by Cancer Council Victoria. Cases and their vital status were ascertained through the Victorian Cancer Registry and the Australian Institute of Health and Welfare, including the National Death Index and the Australian Cancer Database. **MYBRCA** is funded by research grants from the Wellcome Trust (v203477/Z/16/Z), the Malaysian Ministry of Higher Education (UM.C/HlR/MOHE/06) and Cancer Research Malaysia. The Familial Breast Cancer Registry (**OFBCR**) was supported by grant U01CA164920 from the USA National Cancer Institute of the National Institutes of Health. Genotyping for **PLCO** was supported by the Intramural Research Program of the National Institutes of Health, NCI, Division of Cancer Epidemiology and Genetics. The PLCO is supported by the Intramural Research Program of the Division of Cancer Epidemiology and Genetics and supported by contracts from the Division of Cancer Prevention, National Cancer Institute, National Institutes of Health. The **SASBAC** study was supported by funding from the Agency for Science, Technology and Research of Singapore (A*STAR), the US National Institute of Health (NIH) and the Susan G. Komen Breast Cancer Foundation. **SEARCH** is funded by Cancer Research UK [C490/A10124, C490/A16561] and supported by the UK National Institute for Health Research Biomedical Research Centre at the University of Cambridge. The University of Cambridge has received salary support for PDPP from the NHS in the East of England through the Clinical Academic Reserve. **SGBCC** is funded by the National Research Foundation Singapore, NUS start-up Grant, National University Cancer Institute Singapore (NCIS) Centre Grant, Breast Cancer Prevention Programme, Asian Breast Cancer Research Fund and the NMRC Clinician Scientist Award (SI Category). **SKKDKFZS** is supported by the DKFZ. NM was funded by Cancer Research UK (grant number PPRPGM-Nov20\100002)

We thank all the individuals who took part in these studies and all the researchers, clinicians, technicians and administrative staff who have enabled this work to be carried out. ABCS thanks the Blood bank Sanquin, The Netherlands. The ACP study wishes to thank the participants in the Thai Breast Cancer study. Special thanks also go to the Thai Ministry of Public Health (MOPH), doctors and nurses who helped with the data collection process. Finally, the study would like to thank Dr Prat Boonyawongviroj, the former Permanent Secretary of MOPH and Dr Pornthep Siriwanarungsan, the former Department Director-General of Disease Control who have supported the study throughout. The BREOGAN study would not have been possible without the contributions of the following: Manuela Gago-Dominguez, Jose Esteban Castelao, Angel Carracedo, Victor Muñoz Garzón, Alejandro Novo Domínguez, Maria Elena Martinez, Sara Miranda Ponte, Carmen Redondo Marey, Maite Peña Fernández, Manuel Enguix Castelo, Maria Torres, Manuel Calaza (BREOGAN), José Antúnez, Máximo Fraga and the staff of the Department of Pathology and Biobank of the University Hospital Complex of Santiago-CHUS, Instituto de Investigación Sanitaria de Santiago, IDIS, Xerencia de Xestion Integrada de Santiago-SERGAS; Joaquín González-Carreró and the staff of the Department of Pathology and Biobank of University Hospital Complex of Vigo, Instituto de Investigacion Biomedica Galicia Sur, SERGAS, Vigo, Spain. CCGP thanks Styliani Apostolaki, Anna Margiolaki, Georgios Nintos, Maria Perraki, Georgia Saloustrou, Georgia Sevastaki, Konstantinos Pompodakis. CGPS thanks staff and participants of the Copenhagen General Population Study. For the excellent technical assistance: Dorthe Uldall Andersen, Maria Birna Arnadottir, Anne Bank, Dorthe Kjeldgård Hansen. The Danish Cancer Biobank is acknowledged for providing infrastructure for the collection of blood samples for the cases. COLBCCC thanks all patients, the physicians Justo G. Olaya, Mauricio Tawil, Lilian Torregrosa, Elias Quintero, Sebastian Quintero, Claudia Ramírez, José J. Caicedo, and Jose F. Robledo, and the technician Michael Gilbert for their contributions and commitment to this study. PROCAS thanks NIHR for funding. The GENICA Network: Dr. Margarete Fischer-Bosch-Institute of Clinical Pharmacology, Stuttgart, and University of Tübingen, Germany [Hiltrud Brauch, Reiner Hoppe, Wing-Yee Lo], Department of Internal Medicine, Johanniter GmbH Bonn, Johanniter Krankenhaus, Bonn, Germany [YDK, Christian Baisch], Institute of Pathology, University of Bonn, Germany [Hans-Peter Fischer], Molecular Genetics of Breast Cancer, Deutsches Krebsforschungszentrum (DKFZ), Heidelberg, Germany [UH], Institute for Prevention and Occupational Medicine of the German Social Accident Insurance, Institute of the Ruhr University Bochum (IPA), Bochum, Germany [Thomas Brüning, Beate Pesch, Sylvia Rabstein, Anne Lotz]; and Institute of Occupational Medicine and Maritime Medicine, University Medical Center Hamburg-Eppendorf, Germany [Volker Harth]. HUBCS thanks Darya Prokofyeva and Shamil Gantsev. KARMA and SASBAC thank the Swedish Medical Research Counsel. KBCP thanks Eija Myöhänen. MARIE thanks Petra Seibold, Nadia Obi, Sabine Behrens, Ursula Eilber and Muhabbet Celik. MASTOS thanks all the study participants and express appreciation to the doctors: Yiola Marcou, Eleni Kakouri, Panayiotis Papadopoulos, Simon Malas and Maria Daniel, as well as to all the nurses and volunteers who provided valuable help towards the recruitment of the study participants. The MCCS was made possible by the contribution of many people, including the original investigators, the teams that recruited the participants and continue working on follow-up, and the many thousands of Melbourne residents who continue to participate in the study. MYBRCA thanks study participants and research staff (particularly Patsy Ng, Nurhidayu Hassan, Yoon Sook-Yee, Daphne Lee, Lee Sheau Yee, Phuah Sze Yee and Norhashimah Hassan) for their contributions and commitment to this study. The OFBCR thanks Teresa Selander, Nayana Weerasooriya and Steve Gallinger. We thank the SEARCH and EPIC teams. SGBCC thanks the participants and all research coordinators for their excellent help with recruitment, data and sample collection. SKKDKFZS thanks all study participants, clinicians, family doctors, researchers and technicians for their contributions and commitment to this study.

97.          Castro-Mondragon, J. A. *et al.* JASPAR 2022: the 9th release of the open-access database of transcription factor binding profiles. *Nucleic Acids Res* **50**, D165–D173 (2022).

98.          Chen, D. *et al.* Human Primordial Germ Cells Are Specified from Lineage-Primed Progenitors. *Cell Rep* **29**, 4568-4582.e5 (2019).

99.          Chen, D. *et al.* The TFAP2C-Regulated OCT4 Naive Enhancer Is Involved in Human Germline Formation. *Cell Rep* **25**, 3591-3602.e5 (2018).

100.        Boix, C. A., James, B. T., Park, Y. P., Meuleman, W. & Kellis, M. Regulatory genomic circuitry of human disease loci by integrative epigenomics. *Nature* **590**, 300–307 (2021).

101.        Lou, S. *et al.* TopicNet: a framework for measuring transcriptional regulatory network change. *Bioinformatics* **36**, I474–I481 (2020).

102.        Altman, D. G. & Bland, J. M. How to obtain the P value from a confidence interval. *BMJ* **343**, d2304–d2304 (2011).

103.        Langmead, B. & Salzberg, S. L. Fast gapped-read alignment with Bowtie 2. *Nat Methods* **9**, 357–359 (2012).

104.        Heinz, S. *et al.* Simple combinations of lineage-determining transcription factors prime cis-regulatory elements required for macrophage and B cell identities. *Mol Cell* **38**, 576–589 (2010).

105.        Ernst, J. & Kellis, M. ChromHMM: automating chromatin-state discovery and characterization. *Nat Methods* **9**, 215–216 (2012).

106.        Quinlan, A. R. & Hall, I. M. BEDTools: a flexible suite of utilities for comparing genomic features. *Bioinformatics* **26**, 841–842 (2010).

107.        Layer, R. M. *et al.* GIGGLE: a search engine for large-scale integrated genome analysis. *Nat Methods* **15**, 123–126 (2018).
