# Supplementary material for: Genetic links between ovarian ageing, cancer risk and de novo mutation rates
Source: Nature. 2024 Sep 11;633(8030):608–14. doi: 10.1038/s41586-024-07931-x (PMC11410666; doi:10.1038/s41586-024-07931-x)
Supplement: Supplementary file 2 — Reporting Summary [file 41586_2024_7931_MOESM2_ESM.pdf]

nature portfolio

Corresponding author(s): Anna Murray & John Perry

Last updated by author(s): Aug 6, 2024

Reporting Summary

Nature Portfolio wishes to improve the reproducibility of the work that we publish. This form provides structure for consistency and transparency in reporting. For further information on Nature Portfolio policies, see our Editorial Policies and the Editorial Policy Checklist.

Statistics

For all statistical analyses, confirm that the following items are present in the figure legend, table legend, main text, or Methods section.

n/a	Confirmed	
☐	☒	The exact sample size (*n*) for each experimental group/condition, given as a discrete number and unit of measurement
☒	☐	A statement on whether measurements were taken from distinct samples or whether the same sample was measured repeatedly
☐	☒	The statistical test(s) used AND whether they are one- or two-sided<br>*Only common tests should be described solely by name; describe more complex techniques in the Methods section.*
☐	☒	A description of all covariates tested
☐	☒	A description of any assumptions or corrections, such as tests of normality and adjustment for multiple comparisons
☐	☒	A full description of the statistical parameters including central tendency (e.g. means) or other basic estimates (e.g. regression coefficient) AND variation (e.g. standard deviation) or associated estimates of uncertainty (e.g. confidence intervals)
☐	☒	For null hypothesis testing, the test statistic (e.g. *F*, *t*, *r*) with confidence intervals, effect sizes, degrees of freedom and *P* value noted<br>*Give P values as exact values whenever suitable.*
☒	☐	For Bayesian analysis, information on the choice of priors and Markov chain Monte Carlo settings
☒	☐	For hierarchical and complex designs, identification of the appropriate level for tests and full reporting of outcomes
☐	☒	Estimates of effect sizes (e.g. Cohen's *d*, Pearson's *r*), indicating how they were calculated

Our web collection on statistics for biologists contains articles on many of the points above.

Software and code

Policy information about availability of computer code

Data collection	No data was collected
Data analysis	The variant annotation was performed using the ENSEMBL Variant Effect Predictor (VEP) v104<br>Rare variant association testing was performed in BOLT-LMM v2.3. and REGENIEv2.2.4<br>De novo mutations (DNMs) were called using the Platypus variant caller. The commit version used: bayesiandenovofilter.py python script: 414ca566f8b2269d0caae726b21f87e03783ca1a (latest at time of analysis).

For manuscripts utilizing custom algorithms or software that are central to the research but not yet described in published literature, software must be made available to editors and reviewers. We strongly encourage code deposition in a community repository (e.g. GitHub). See the Nature Portfolio guidelines for submitting code & software for further information.

Data

Policy information about availability of data

All manuscripts must include a data availability statement. This statement should provide the following information, where applicable:

- Accession codes, unique identifiers, or web links for publicly available datasets
- A description of any restrictions on data availability
- For clinical datasets or third party data, please ensure that the statement adheres to our policy

All data used in discovery analyses are available upon application from the UK Biobank study and Genomics England. Research on the de-identified patient data

used in this publication can be carried out in the Genomics England Research Environment subject to a collaborative agreement that adheres to patient led governance. All interested readers will be able to access the data in the same manner that the authors accessed the data. For more information about accessing the data, interested readers may contact research-network@genomicsengland.co.uk or access the relevant information on the Genomics England website: https://www.genomicsengland.co.uk/research.

The deCODE dataset was used for replication purposes and only summary level results for the specific findings are provided.

All datasets are available through an application process to individual studies.

Research involving human participants, their data, or biological material

Policy information about studies with human participants or human data. See also policy information about sex, gender (identity/presentation), and sexual orientation and race, ethnicity and racism.

Reporting on sex and gender	The study was restricted to participants of female sex as defined by genetic markers, as only biological females undergo menopause, the trait being investigated.
Reporting on race, ethnicity, or other socially relevant groupings	We restricted our analyses to individuals of European ancestry as defined by genetic principle component analysis.
Population characteristics	Data from participants aged 40-70 from the UK Biobank were included in our analyses. We did not include disease or treatment characteristics as covariates.
Recruitment	Volunteers to UK Biobank
Ethics oversight	UK Biobank has ethics approval from the North West MREC under application number 21/NW/0157

Note that full information on the approval of the study protocol must also be provided in the manuscript.

Field-specific reporting

Please select the one below that is the best fit for your research. If you are not sure, read the appropriate sections before making your selection.

[x] Life sciences   [ ] Behavioural & social sciences   [ ] Ecological, evolutionary & environmental sciences

For a reference copy of the document with all sections, see nature.com/documents/nr-reporting-summary-flat.pdf

Life sciences study design

All studies must disclose on these points even when the disclosure is negative.

Sample size	All genetic females with natural age at menopause in Uk Biobank
Data exclusions	None
Replication	Replication was performed using two study populations - the Icelandic deCODE study and the BRIDGES study. We were able to replicate the novel genetic associations with ANM , but we were not able to replicate the de novo mutation discovery from 100KGP in deCODE
Randomization	The principle exposure in this study is naturally occurring genetic variants, meaning that we were unable to randomise the individuals in the study. To account for possible confounding we used a linear mixed model and adjusted for technical and demographic covariates.
Blinding	Blinding is not applicable to this study, as it is a genome-wide study of rare coding variants and not a randomised controlled trial. We did not deliver any intervention to participants in this study.

Reporting for specific materials, systems and methods

We require information from authors about some types of materials, experimental systems and methods used in many studies. Here, indicate whether each material, system or method listed is relevant to your study. If you are not sure if a list item applies to your research, read the appropriate section before selecting a response.

Materials & experimental systems

n/a	Involved in the study
☒	☐ Antibodies
☒	☐ Eukaryotic cell lines
☒	☐ Palaeontology and archaeology
☒	☐ Animals and other organisms
☒	☐ Clinical data
☒	☐ Dual use research of concern
☒	☐ Plants

Methods

n/a	Involved in the study
☒	☐ ChIP-seq
☒	☐ Flow cytometry
☒	☐ MRI-based neuroimaging

Plants

Seed stocks

Report on the source of all seed stocks or other plant material used. If applicable, state the seed stock centre and catalogue number. If plant specimens were collected from the field, describe the collection location, date and sampling procedures.

Novel plant genotypes

Describe the methods by which all novel plant genotypes were produced. This includes those generated by transgenic approaches, gene editing, chemical/radiation-based mutagenesis and hybridization. For transgenic lines, describe the transformation method, the number of independent lines analyzed and the generation upon which experiments were performed. For gene-edited lines, describe the editor used, the endogenous sequence targeted for editing, the targeting guide RNA sequence (if applicable) and how the editor was applied.

Authentication

Describe any authentication procedures for each seed stock used or novel genotype generated. Describe any experiments used to assess the effect of a mutation and, where applicable, how potential secondary effects (e.g. second site T-DNA insertions, mosiacism, off-target gene editing) were examined.
